# The future health and economic burden of obesity-attributable type 2 diabetes and liver disease among the working-age population in Saudi Arabia

**DOI:** 10.1371/journal.pone.0271108

**Published:** 2022-07-14

**Authors:** Timothy Coker, Jennifer Saxton, Lise Retat, Khalid Alswat, Suliman Alghnam, Rajaa Mohammad Al-Raddadi, Habeeb Ibrahim Abdul Razack, Laura Webber, Saleh A. Alqahtani

**Affiliations:** 1 HealthLumen Ltd, London, United Kingdom; 2 Liver Disease Research Centre, Department of Medicine, College of Medicine, King Saud University, Riyadh, Saudi Arabia; 3 Population Health Department, King Abdullah International Medical Research Centre, King Saud Bin Abdulaziz University for Health Sciences, King Abdulaziz Medical City, National Guard Health Affairs, Riyadh, Saudi Arabia; 4 Saudi Diabetes Study Research Group, King Fahd Medical Research Center, King Abdulaziz University, Jeddah, Saudi Arabia; 5 Department of Community Medicine, Faculty of Medicine, King Abdulaziz University, Jeddah, Saudi Arabia; 6 Faculty of Medicine & Health Sciences, Universiti Putra Malaysia, Sedang, Selangor, Malaysia; 7 Department of Cardiac Sciences, College of Medicine, King Saud University, Riyadh, Saudi Arabia; 8 Division of Gastroenterology & Hepatology, Johns Hopkins University, Baltimore, MD, United States of America; 9 Liver Transplant Centre, King Faisal Specialist Hospital & Research Centre, Riyadh, Saudi Arabia; University of York, UNITED KINGDOM

## Abstract

**Background:**

Obesity and type 2 diabetes (T2DM) are increasing in Saudi Arabia (SA). Among other conditions, these risk factors increase the likelihood of non-alcoholic fatty liver disease (NAFLD), which in turn increases risks for advanced liver diseases, such as non-alcoholic steatohepatitis (NASH), cirrhosis and cancer. The goal of this study was to quantify the health and economic burden of obesity-attributable T2DM and liver disease in SA.

**Methods:**

We developed a microsimulation of the SA population to quantify the future incidence and direct health care costs of obesity-attributable T2DM and liver disease, including liver cancer. Model inputs included population demographics, body mass index, incidence, mortality and direct health care costs of T2DM and liver disease and relative risks of each condition as a function of BMI category. Model outputs included age- and sex-disaggregated incidence of obesity-attributable T2DM and liver disease and their direct health care costs for SA’s working-age population (20–59 years) between 2020 and 2040.

**Results:**

Between 2020 and 2040, the available data predicts 1,976,593 [± 1834] new cases of T2DM, 285,346 [±874] new cases of chronic liver diseases, and 2,101 [± 150] new cases of liver cancer attributable to obesity, amongst working-age people. By 2040, the direct health care costs of these obesity-attributable diseases are predicted to be 127,956,508,540 [± 51,882,446] USD.

**Conclusions:**

The increase in obesity-associated T2DM and liver disease emphasises the urgent need for obesity interventions and strategies to meaningfully reduce the future health and economic burden of T2DM, chronic liver diseases and liver cancer in SA.

## Introduction

### Background

Obesity, defined as a body mass index (BMI) ≥30 in non-Asian populations, is a major risk factor for non-communicable diseases (NCDs). National estimates of Saudi Arabia (SA) from 2013 indicate that 24.1% of men and 33.5% of women were obese [1]. Obesity contributes to the development of several chronic diseases, including type 2 diabetes (T2DM) [2] and non-alcoholic fatty liver disease (NAFLD) [3]. There was a 73.5% global increase in T2DM between 1990 and 2017, attributable to high BMI [4]. T2DM creates an additional route to liver disease, potentially more than doubling the risk of developing advanced liver disease [5]. A high prevalence of NAFLD amongst T2DM patients has also been identified, where nearly two-thirds of patients receiving liver biopsies had developed the more severe form, non-alcoholic steatohepatitis (NASH) [6]. Notably, there is an increased risk of developing T2DM even with a BMI as low as 21 kg/m^2^ [7].

NAFLD is becoming one of the most common liver diseases worldwide, affecting around one-quarter of the world population, with the highest prevalence of NAFLD reported from the Middle East (32%) and South America (30%) [8]. Initially asymptomatic, 10–30% of cases progress to NASH, which can lead to cirrhosis, hepatocellular carcinoma (HCC), and the need for liver transplant [6, 9]. Cirrhosis is the leading cause of liver-related mortality globally, and prevalence of irreversible cirrhosis due to NASH more than tripled between 1990 and 2017, with rising levels of obesity and metabolic syndrome identified as plausible reasons [10]. If recognised early, NAFLD and NASH can be treated through weight-loss interventions and modification of other metabolic risk factors such as dyslipidemia, thus reducing the risk of cirrhosis [11].

In SA, NAFLD has overtaken hepatitis-induced liver disease as the main indication for liver transplants [12]. Challenges in diagnosis and surveillance of NAFLD and NASH, including patient unawareness or neglect, missed diagnosis, lack of or absence of symptoms, mean that estimates of the population burden of obesity-related liver disease are limited to a few studies [13, 14]. One hospital-based study found 22.5% of patients with chronic liver disease had NAFLD [15]. In 2017, SA reported incidence and mortality rates from NASH-related cirrhosis as 10.5 and 1.2 per 100,000 population [16]. There was also a 25% increase in cirrhosis deaths between 2007 and 2017, with cirrhosis ranked 10th as a cause of premature death [17]. This is worrying, in particular for the working age population, as liver disease is one of the major causes of working years of life lost [18]. Despite this, there lacks a broad, quantitative picture of the current and future burden of liver diseases on health and economic outcomes amongst the working age population in SA [18].

Projections of future obesity-attributable liver disease are helpful for policymakers to anticipate health system demand, budgeting, and to devise interventions to offset concerning trends. A recent Markov model projected trends in NAFLD, NASH, and their related outcomes up to 2030, where no estimates previously existed for SA. Authors estimated over 12 million NAFLD cases, doubled prevalent rates of compensated cirrhosis and advanced liver disease, and annual incident liver deaths may rise to 4800 deaths by 2030 [17]. However, the drawbacks included the Markovian approach taken, since it relies on cohorts, which creates overly homogenous hypothetical populations. The Markov principle limits the model’s ability to account for individual risk factor history, meaning results could be less reflective of SA’s population. There was also no consideration of associated health care costs.

### Rationale

Given the recent upward trend in obesity, concomitant trends in T2DM and liver disease, as well as a downward trend in physical activity [19] and the related healthcare costs associated with this disease burden, it is critical to understand how the burden will unfold for future healthcare planning. Knowing the extent of the year-on-year burden will act as a lever for change and encourage the implementation of cost-effective interventions to prevent or reduce obesity and reverse trends in related NCDs [11, 20].

### Aims

In this study, we developed a microsimulation model to assess the projected health and economic burden of obesity-attributable diseases in SA amongst the working-age population (20–59 years) between 2020 and 2040, by age and sex, with the aim of estimating the:

1. Obesity-attributable incidence of T2DM, chronic liver diseases and liver cancer
2. Direct health care costs of obesity-attributable T2DM, chronic liver diseases and liver cancer

## Methods

### Model overview

Microsimulation models create more realistic hypothetical populations and are referred to as the ‘best method for risk factor and chronic disease modelling’ by the Organisation for Economic Co-operation and Development [21]. Findings from microsimulations are frequently used by policymakers to make clear decisions about funding and planning of future interventions and prevention strategies [22]. A 2014 study modelled future disease trends in 53 European countries, supporting policymakers’ decisions in resource allocation and interventions [23]. The study findings advocate the need to have more effective policies for obesity.

The microsimulation model used in this study was originally developed for the United Kingdom (UK) Obesity Foresight Report [24]. It has since been adapted to calculate health and economic projections for several chronic diseases resulting from a range of risk factors in over 70 countries, including the Middle East [20, 25].

**Fig 1:**
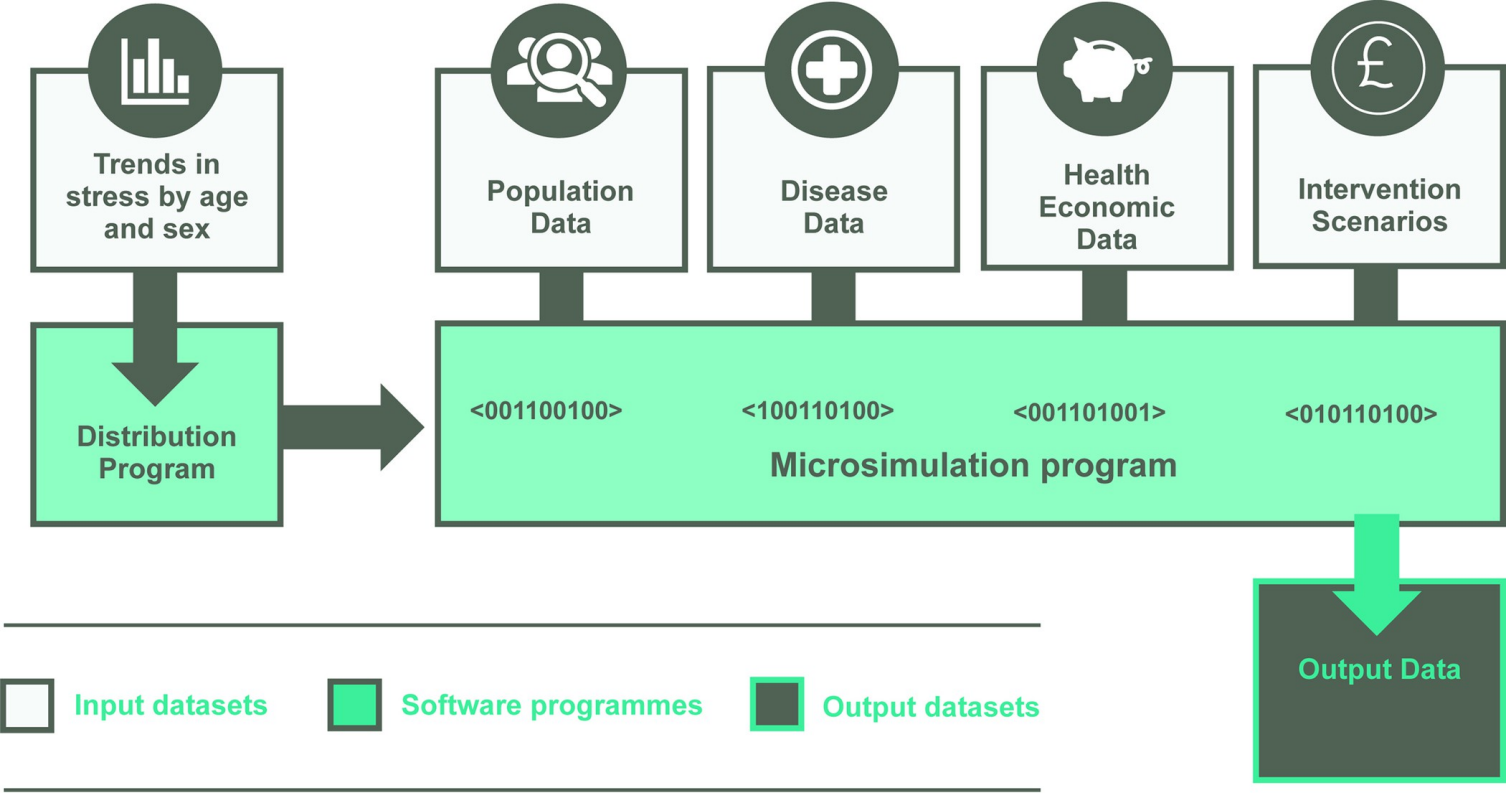
Overview of the microsimulation model.

At the input-level, the program requires 1) risk factor data following trends by age and sex over time; 2) demographic data to create a hypothetical population of individuals living now and in the future; 3) epidemiological disease data including relative risks (RRs) quantifying associations between risk factors and diseases; 4) health economic data on the financial burden of disease; and 5) specification of a hypothetical future scenario. The population is dynamic, permitting individual births, deaths and experience of the disease. Each individual’s disease status can change according to its natural course, including through different stages of liver fibrosis, which may progress and regress over time. The model is depicted in Fig 1.

### Model specification

For the purpose of this study, chronic liver diseases include the International Classification of Disease (ICD) version 10 category: ‘cirrhosis and other chronic liver diseases’, which includes NAFLD and NASH, but excludes hepatocellular carcinoma, as followed in the Global Burden of Disease (GBD) data.

Input data included 1) demographic (age, sex, births, deaths, fertility rates and projected population); 2) risk factor (BMI category—healthy weight <25.00 kg/m^2^, pre-obese 25.00–29.99 kg/m^2^, obese ≥30.00 kg/m^2^ based on World Health Organisation standards, by age and sex); 3) disease epidemiology (T2DM, chronic liver diseases, liver cancer); and 4) direct health care costs of diseases.

We ran the model from 2020 to 2040. We simulated 100 million individuals, the number being a compromise between minimising the Monte Carlo error and the run time of the microsimulation. BMI trends, projected using an age- and sex-stratified non-linear multivariate categorical regression model, were used to assign BMI to individuals, who contracted the modelled diseases with a particular probability according to their BMI, age and sex. Together with disease mortality and health care cost data, we estimated disease incidence and health care costs over the modelled period. To calculate obesity-attributable outcomes, we ran an additional scenario in which the prevalence of obesity and pre-obesity was 0%, the outcomes of which were subtracted from the current trend scenario.

### Literature reviews: Input data

A more detailed description of our literature review is given in S1 Table.

We conducted focused literature reviews in Pubmed and Google to identify suitable peer-reviewed and grey literature. For BMI/obesity and each disease, we first searched for sex- and age-disaggregated incidence, prevalence and mortality data, then for RRs for each disease by BMI category, prioritising adjusted RRs if adjusted and unadjusted estimates were available.

### Risk factor: BMI/Obesity

No longitudinal age- and sex-stratified BMI data were available for SA, so cross-sectional data from 2005 [26], 2011 [27], 2013 [1], and 2016 [28] were used. We excluded people aged 50+ from the 2016 data, for both sexes, due to very small sample sizes.

### Disease epidemiology and cost data

A summary of disease epidemiology and cost data is presented in Table 1. We were unable to find suitable RRs from SA or the Middle East and Gulf, so we used alternatives. Appropriate cost data were not available for SA, so we used data from the United Kingdom, having well established and similar health care infrastructure as that of SA.

**Table 1:** Data sources for disease epidemiology and cost data used in the microsimulation.

Disease / measure	Source	Notes
**Type 2 Diabetes**		
Incidence	Global Burden of Disease (GBD), 2017 [16]	• Stratified by age and sex
Relative risk (RR) as a function of Body Mass Index (BMI) (to the nearest 1)^a^	Vazquez et al., 2007 [29]	• Estimates are for Asian males and females.
Costs	Public Health England (PHE), 2020 [30]	• Annual mean health care expenditure for UK patients with diabetes, consisting of costs for primary and secondary care and prescription costs, of 2,704.41 USD^b^ per patient
**Chronic liver diseases**		
Incidence, prevalence, mortality	GBD, 2017 [16]	• Stratified by age and sex; GBD disease code “cirrhosis and other chronic liver diseases” (excluding liver cancer).
RR as a function of BMI (to the nearest 1)^a^	Harris et al., 2019 [31]	• Estimates are an age, gender, and ethnicity-adjusted odds ratio (OR) for the UK. • Due to <10% incidence, we applied the rare disease assumption and assumed ORs were equal to RRs [32].
Costs	PHE, 2020 [30]	• Annual mean health care expenditure for UK patients with chronic liver diseases, consisting of costs for primary and secondary care and prescription costs, of 8,923.62 USD^b^ per patient
**Liver cancer**		
Incidence	Saudi Cancer Registry, 2014 [33]	• Stratified by age and sex
Mortality	Global Cancer Observatory, 2018 [34]	• Stratified by age and sex
RR as a function of BMI category^a^	Chen et al., 2012 [35]	• Global meta-analysis • Estimates are for Asian populations.
Costs	Georghiou et al., 2012 [36]	• Annual health care expenditure, for UK cancer patients in the last 12 months of life, consisting of primary care costs, secondary inpatient and outpatient costs, of 13,259.80 USD^b^ per patient

^a^ Adjusted RRs were prioritised over unadjusted RRs if both estimates were available

^b^ Costs adjusted to 2010 using https://eppi.ioe.ac.uk/costconversion/

### Model outputs

We extracted age- and sex-disaggregated estimates for obesity-attributable incidence of T2DM, liver disease and liver cancer, and direct health care costs at five-yearly intervals (2020–2040). We focused our analysis on individuals aged 20–59 as they comprise the vast majority of the working-age population and were the population for which the highest quality data were available in terms of the number of data points and/or sample size. This cohort is also important in terms of policy planning since they make up a substantial part of the workforce. Results were rescaled to the projected population for SA.

## Results

### Cumulative incidence of obesity-attributable diseases

The projected obesity-attributable cumulative incidence of T2DM, chronic liver diseases and liver cancer, for working-age males and females are presented in Table 2. Under current BMI trends, we predict, using the microsimulation model, 1.98 million cases of T2DM, over 285,000 cases of chronic liver diseases and 2101 cases of liver cancer attributable to obesity by 2040. This represents 92.2%, 70.6% and 23.7% of all cases, respectively. By 2040, we predict a marked difference by sex as males could account for 69%, 70% and 72% of the cumulative burden of obesity-attributable T2DM, liver disease and liver cancer, respectively.

**Table 2:** Obesity-attributable incidence of type 2 diabetes, chronic liver diseases and liver cancer amongst working-age males and females (20–59 years) in the Saudi Arabian population, 2020–2040.

	Cumulative incidence [±95% confidence limits]				
Disease	2020	2025	2030	2035	2040
**Type 2 Diabetes**					
Males	55,831 [± 297]	353,589 [± 761]	675,815 [± 1068]	1,016,338 [± 1329]	1,364,328 [± 1557]
Females	27,676 [± 202]	170,597 [± 503]	319,548 [± 692]	469,409 [± 844]	612,265 [± 971]
Total	83,507 [± 359]	524,186 [± 912]	995,363 [± 1273]	1,485,748 [± 1574]	1,976,593 [± 1834]
**Chronic Liver Diseases** ^**b**^					
Males	8,988 [± 154]	54,420 [± 383]	101,677 [± 526]	150,326 [± 643]	199,564 [± 743]
Females	4,258 [± 99]	25,605 [± 245]	46,692 [± 332]	66,988 [± 402]	85 783 [± 460]
Total	13 246 [± 184]	80 025 [± 455]	148 369 [± 623]	217 314 [± 758]	285 346 [± 874]
**Liver Cancer**					
Males	47 [± 21]	346 [± 58]	720 [± 85]	1111 [± 107]	1506 [± 129]
Females	17 [± 12]	117 [± 32]	262 [± 49]	425 [± 63]	595 [± 76]
Total	63 [± 25]	463 [± 66]	982 [± 98]	1,535 [± 125]	2,101 [± 150]
**Combined Diseases of Interest**					
Males	64,866 [± 336]	408,355 [± 854]	778,212 [± 1194]	1,167,775 [± 1480]	1,565,398 [± 1730]
Females	31,950 [± 225]	196,319 [± 561]	366,503 [± 769]	536,822 [± 937]	698,642 [± 1077]
Total	96,816 [± 404]	604,674 [± 1021]	1,144,715 [± 1 420]	1,704,597 [± 1752]	2,264,041 [± 2038]

^a^ errors around the projections reflect the accuracy of the microsimulation; they do not represent 95% confidence intervals

^b^ Liver disease includes the following International Classification of Disease version 10 category: ‘cirrhosis and other chronic liver diseases’, and excludes hepatocellular carcinoma

Additional data outputs are presented in S2–S5 Tables. Sex and age-group disaggregated cumulative incidence estimates are available in S2 Table. These data suggest wide variations by sex and age, though generally, the greatest burden is in middle and older age groups (35 years+) and males.

The projected obesity-attributable annual incidence of these diseases is presented in S3 Table. For obesity-attributable T2DM, the annual incidence per 100,000 is expected to rise from 239.9 [± 1.0] in 2020 to 245.2 [± 1.0] in 2030, before falling to 230.8 [± 1.0] in 2040. Based on projected growth in the SA population, the total number of new cases per year is predicted to increase 17.4% from 83,507 [± 359] in 2020 to 98,045 [± 422] in 2040.

For chronic liver diseases, obesity-attributable annual incidence per 100,000 is similarly predicted to decrease from 38.0 [± 0.5] in 2020 to 31.7 [± 0.5] in 2040, but due to population growth this represents a 1.8% increase from 13,246 [± 184] total cases in 2020 to 13,478 [± 194] in 2040.

### Cumulative direct health care costs of obesity-attributable diseases

Obesity-attributable cumulative health care costs of T2DM, chronic liver diseases and liver cancer amongst working-age males and females in SA are presented in Table 3. Costs are further disaggregated by sex- and age-group in S2 Table. Annual costs are provided in S4 Table. By 2040, cumulative costs of obesity-attributable T2DM amongst working-age adults are predicted to exceed 84.4 billion USD. Health care costs of obesity-attributable chronic liver diseases and liver cancer are predicted to exceed 43 billion USD and 50 million USD, respectively. This accounts for 63.4%, 42.3% and 14.6% of the costs for these diseases, respectively. Males account for 65.4% of obesity-attributable costs, reflecting the greater disease incidence amongst males for all three diseases.

**Table 3:** Obesity-attributable health care costs between 2020 and 2040 amongst the Saudi Arabian working population (20–59 years).

	Cumulative Health Care Costs in USD [±95% confidence limits]				
Disease	2020	2025	2030	2035	2040
**Type 2 Diabetes**					
Males	1,459,819,285 [± 4,311,608]	10,838,449,380 [± 10,727,418]	23,333,273,316 [± 14,685,972]	38,290,983,763 [± 17,844,821]	54,753,621,938 [± 20,485,258]
Females	817,655,103 [± 3,100,498]	5,997,136,758 [± 7,700,790]	12,876,910,602 [± 10,547,019]	21,004,438,952 [± 12,784,834]	29,675,572,997 [± 14,593,395]
Total	2,277,474,389 [± 5,310,655]	16,835,586,138 [± 13,205,289]	36,210,183,917 [± 18,080,857]	59,295,422,715 [± 21,951,985]	84,429,194,935 [± 25,151,799]
**Chronic Liver Diseases** ^**a**^					
Males	812,608,022 [± 7,689,576]	5,915,514,096 [± 19,238,787]	12,570,103,910 [± 26,461,407]	20,416,514,582 [± 32,286,461]	28,900,053,383 [± 37,214,807]
Females	415,115,894 [± 5,285,827]	3,024,787,087 [± 13,234,683]	6,443,864,454 [± 18,263,168]	10,420,669,017 [± 22,325,182]	14,576,908,694 [± 25,730,502]
Total	1,227,723,916 [± 9,331,107]	8,940,301,183 [± 23,351,398]	19,013,968,364 [± 32,151,973]	30,837,183,599 [± 39,253,399]	43,476,962,077 [± 45,243,791]
**Liver Cancer**					
Males	940,472 [± 570,743]	7,023,220 [± 1,453,560]	14,838,899 [± 2,050,578]	23,384,736 [± 2,546,141]	32,216,649 [± 2,988,104]
Females	534,790 [± 312,212]	3,776,661 [± 811,500]	8,059,809 [± 1,177,090]	12,873,426 [± 1,509,082]	18,134,879 [± 1,801,602]
Total	1,475,262 [± 650,556]	10,799,881 [± 1,664,743]	22,898,708 [± 2,364,405]	36,258,162 [± 2,959,757]	50,351,528 [± 3,489,203]
**All Diseases**					
Males	2,273,367,779 [± 8,834,325]	16,760,986,696 [± 22,075,354]	35,918,216,125 [± 30,332,964]	58,730,883,080 [± 36,977,507]	83,685,891,970 [± 42,585,402]
Females	1,233,305,788 [± 6,136,003]	9,025,700,506 [± 15,333,543]	19,328,834,864 [± 21,122,700]	31,437,981,395 [± 25,770,974]	44,270,616,570 [± 29,635,649]
Total	3,506,673,566 [± 10,756,200]	25,786,687,202 [± 26,878,222]	55,247,050,989 [± 36,962,916]	90,168,864,475 [± 45,071,933]	127,956,508,540 [± 51,882,446]

Errors around the projections reflect the accuracy of the microsimulation; they do not represent 95% confidence intervals

^a^ Liver disease includes the following International Classification of Disease version 10 category: ‘cirrhosis and other chronic liver diseases’, and excludes hepatocellular carcinoma

The greatest costs are seen for middle and older aged people in our cohort, consistent with the greater disease incidence amongst these ages. For males, the greatest overall cumulative cost burden by 2040 is accounted for by those 45–49 and 50–54 years, equalling 15.9 billion USD and 15.3 billion USD, respectively. For females, the same two age groups are associated with the greatest health care costs (8.4 billion USD for 45–49 years; 7.8 billion USD for 50–54 years).

## Discussion

### Key findings

The developed model predicts 2.26 million new cases of T2DM, chronic liver diseases and liver cancer in SA by 2040 that will be attributable to obesity. Importantly, the model highlights marked sex and age differences, with males and those ≥35 years being more frequently affected.

It is unsurprising that older people account for the greatest burden of obesity-attributable liver disease in our microsimulation. Chronic liver disease, such as NAFLD develop slowly. In one European study, the average age at diagnosis was 54–57 years [37]. Liver cancer has a relatively lower incident rate in SA, with a mean age at diagnosis of >60, which could explain the low number of predicted cases in our study [38].

In general, we found a greater disease burden amongst males. This is likely a consequence of the generally higher disease incidence amongst males in our inputs (for liver disease), and a projected reduction in BMI amongst females versus increases amongst males >34 years (S5 Table). Of concern is that one-third of males are predicted to become diabetic before age 40; there are limited data available to understand whether this represents an increase in the proportion of younger males developing T2DM compared to current levels. The burden to individuals and health systems in SA in general from T2DM is considerable, especially for those diagnosed at a young age who will live with the disease for longer. This will not only impact healthcare costs but also have important implications for businesses as the working population are affected.

Obesity-attributable T2DM and liver disease are predicted to result in healthcare costs of nearly $128 billion USD by 2040, an average of over $6 billion USD per year. According to the latest data, this represents a substantial proportion of SA’s health care spending (~17.8%, based on a healthcare expenditure of 35.9 million USD in 2017) [39]. Tackling obesity and obesity-related liver disease would therefore be highly influential in preventing future costs to SA’s health system.

### How our results compare with other SA studies?

To our knowledge, this is the first study to assess the future disease burden of obesity-attributable incidence of T2DM in SA.

For obesity-attributable liver disease, the closest, but not directly comparable study used a Markov model for SA, estimating a NAFLD incidence rate of 9.8 per 1,000 population (386,100 cases) for 2030 [17]. Our results for 2030 are substantially lower, showing an obesity-attributable chronic liver diseases incidence rate of 0.57 per 1,000 (13,776 cases). Though there are methodological differences, the discrepancy in findings may be largely explained by our use of GBD data, which reports an incidence of ~0.1–0.3 per 1,000 across working age groups [16]. GBD data are based on ICD-10 codes where possible; otherwise, data are modelled. Given that ICD-10 codes require a diagnosis, and NAFLD is largely undiagnosed until it advances in later years, it is likely the GBD data substantially underestimates the true incidence of obesity-related liver disease. In contrast, Alswat and colleagues used sources which directly measured NAFLD/NASH in sub-national and hospital studies for a wider age range, resulting in a much higher baseline NAFLD prevalence estimate than ours and the GBD’s [17]. A similar discrepancy was noted for liver cancer predictions, with Alswat and colleagues projecting higher numbers of cases. Our restriction to the working-age population also meant the majority of HCC cases would not yet have developed.

### Policy implications

SA has a young population, with more than 60% of people <40 years of age [40]. This represents both a risk and an opportunity. A risk because T2DM is predicted to increasingly affect people from age 35 in our model, and NAFLD has been reported to affect a quarter of adults <35 years [41]. Coupled with increasing life expectancy in SA, there will be an extended time for T2DM and NAFLD to progress towards its more serious complications in older age. The opportunity comes in the availability of obesity-related interventions to prevent and treat T2DM and NAFLD, with ample chance to halt the increase in these obesity-attributable diseases [11]. Our data indicates that males >35 years are a key target group, showing the most concerning trends in obesity-attributable diseases, and who could account for two-thirds of obesity-attributable direct health care costs by 2040.

A further issue arising from our study is the lack of liver disease surveillance system in SA [13]. This presented us with challenges in obtaining comprehensive liver disease and RR data for the microsimulation. For people in SA, poor surveillance could mean a late diagnosis of treatable liver disease, implicating a person’s length and quality of life. A recent report by the Health Policy Partnership set out recommendations to better identify and monitor NASH in the Middle East, including improved data collection, education of primary care providers on NAFLD/NASH, and ‘clear care pathways to encourage more effective diagnosis and high-quality care’ [42]. Relatively simple, non-invasive, point-of-care liver function tests (e.g. transient elastography, FibroScan^®^) could be used in SA’s primary care settings as part of improved surveillance of liver disease, with recent evidence suggesting cost-efficient early detection of fibrosis amongst NAFLD patients [43]. ‘Saudi Vision 2030’ policy initiative aims to envision several transformation programs for the country by improving the quality of life of people, promoting healthy living, and providing efficient healthcare services [44]. The Health Sector Transformation Program of this initiative strategically targets facilitating access to healthcare services, improving the quality and efficiency of health services, and promoting prevention of health risks. Obesity is a major health risk and focus of the program. The data obtained from our microsimulation model may aid and support this initiative to drive its aims forward. Besides, sequential testing strategies based on well-defined referral pathways among primary care and secondary care could provide a cost-effective approach.

### Strengths of our study

Our study is the first to use microsimulation modelling to quantify the future health burden of several obesity-attributable diseases in SA. Our obesity focus is pertinent, considering the regional and global rise in NAFLD. This is also the first paper to provide projections for direct health care costs of obesity-attributable liver disease and T2DM, highlighting to policymakers the importance of prioritising obesity interventions now, so that future costs can be averted.

### Limitations

A key limitation of our study was the lack of high-quality liver disease surveillance data, cost data, and no RRs linking obesity to liver disease outcomes from SA. We relied on some ‘proxy’ data in terms of costs and RRs. As a result, there is likely to be a higher amount of uncertainty around the estimates. However, this highlights the need for improved surveillance systems which will improve the accuracy of the model estimates. Sensitivity analysis would be useful in exploring the impact of input assumptions on predicted liver disease prevalence; this was beyond the scope of this particular project. Indirect costs were not considered due to insufficient data. Secondly, we may have further underestimated obesity-attributable disease by not including the effect of T2DM on liver disease/cancer. Besides, we did not explore different policy scenarios for obesity reduction. Finally, restricting the study to the working-age population, while deliberately highlighting future health impacts on the most economically productive group, means the absolute numbers of liver cancer cases are very small across all model years, so these results should be interpreted with caution.

### Concluding statement

The microsimulation model predicts by 2040 in SA, 2.26 million (9,436 per 100,000) new cases of T2DM, liver disease and liver cancer attributable to obesity, arising to a substantial economic cost. Our findings show that urgent action is needed to implement an effective national policy to address the epidemic of obesity and T2DM. Unless action is taken to effectively prevent and treat obesity, it will continue to be a major driver of associated diseases in SA, including liver disease. Improved liver disease surveillance and obesity monitoring will help refine epidemiological estimates, improve patient care, and guide policymakers to better design obesity policy to avert its future consequences. Further research may focus on estimating the future benefits of reducing obesity on incidence and costs of T2DM and liver disease by comparing different scenarios such as the WHO target to ‘Halt the rise in obesity by 2025’.

## Supporting information

S1 Table Literature review process for input data. (PDF)

S2 Table Projected obesity-attributable disease outcomes and direct healthcare costs, by sex and working age group (2020 to 2040). (PDF)

S3 Table Projected obesity-attributable annual incidence (a) and prevalence (b) of T2DM, chronic liver diseases and liver cancer, by sex and working age group (2020 to 2040).
(PDF)

S4 Table Projected obesity-attributable annual costs of T2DM, chronic liver diseases and liver cancer, by sex and working age group (2020 to 2040; currency USD). (PDF)

S5 Table Projected WHO obesity categories, by sex and working age group (2020 to 2040). (PDF)
