# Supplementary material for: The future health and economic burden of obesity-attributable type 2 diabetes and liver disease among the working-age population in Saudi Arabia
Source: PLoS One. 2022 Jul 14;17(7):e0271108. doi: 10.1371/journal.pone.0271108 (PMC9282435; doi:10.1371/journal.pone.0271108)
Supplement: S1 Table — (PDF) [file pone.0271108.s001.pdf]

S1 Table: Literature review process for input data

General		• We conducted focused literature reviews in PubMed and Google to identify suitable peer-reviewed and grey literature.<br>• For BMI/obesity and each disease, we first searched for sex- and age-disaggregated incidence, prevalence and mortality data, then for RRs for each disease by BMI category, prioritising adjusted RRs if adjusted and unadjusted estimates were available.<br>• Where no suitable data was found for SA, we first prioritised comparable data from the Middle East and Gulf regions, followed by neighbouring regions and countries.
Risk factor: BMI/Obesity		• No longitudinal age- and sex-stratified BMI data were available for SA, so cross-sectional data from 2005, 2011, 2013, and 2016 were used.
		• We excluded people aged 50+ from the 2016 data, for both sexes, due to very small sample sizes.
Disease incidence, prevalence, and mortality	*Incidence*	• For T2DM and chronic liver diseases, we used incidence estimates in SA stratified by age and sex from the GBD study, 2017.<br>• Data for the latter disease were obtained using the disease code "cirrhosis and other chronic liver diseases (excluding liver cancer)".<br>• For liver cancer, we used data stratified by age and sex from the SCR, 2014.
	Prevalence	• For chronic liver diseases, we used prevalence estimates in SA stratified by age and sex from GBD 2017.<br>• Prevalence was converted to incidence using prevalence and mortality data according to WHO DISMOD II equations.
	Mortality	• For chronic liver diseases, we used mortality estimates in SA stratified by age and sex from GBD 2017.<br>• For liver cancer, SA mortality data stratified by age and sex was obtained from the GCO, 2018.
RRs as a function of BMI category		• We were unable to find suitable RRs from SA or the Middle East and Gulf, so we used alternatives.<br>• We prioritised adjusted RRs over unadjusted RRs where both estimates were available.

	T2DM	• Data were taken from Vazquez et al. 2007, which focused on an Asian population.
	Chronic liver diseases	• Data were taken from Harris et al. 2019.<br>• This UK-based study provides OR estimates adjusted for age, sex, and ethnicity.<br>• Due to the incidence of this disease being lower than 10% of the population, we applied the rare disease assumption, assuming that odds ratios are equivalent to RRs.
	Liver cancer	• Data were from Chen et al. 2012. This is a global meta-analysis, from which we selected estimates for Asian populations, as this was deemed most reflective of SA.
Costs	• Appropriate cost data were not available for SA. Data from Egypt was available but costs from the UK were deemed more appropriate given the more similar health care system.<br>• All costs were adjusted to 2010 US dollars using the PPP calculator at EPPI: https://eppi.ioe.ac.uk/costconversion/	

Abbreviations*: BMI, body mass index; DISMOD: disease modelling; GBD, Global Burden of Disease; GCO, Global Cancer Observatory; OR, odds ration; PPP, Purchasing Power Parities; RR, relative risk; SA, Saudi Arabia; SCR, Saudi Cancer Registry; T2DM, type-2 diabetes mellitus; UK, United Kingdom; US, United States; WHO, World Health Organization*
