# Supplementary material for: The future health and economic burden of obesity-attributable type 2 diabetes and liver disease among the working-age population in Saudi Arabia
Source: PLoS One. 2022 Jul 14;17(7):e0271108. doi: 10.1371/journal.pone.0271108 (PMC9282435; doi:10.1371/journal.pone.0271108)
Supplement: S2 Table — (PDF) [file pone.0271108.s002.pdf]

S2 Table: Projected obesity-attributable disease outcomes and direct healthcare costs, by sex and working age group (2020 to 2040)

Indicator, by sex and age	2020	2025	2030	2035	2040
	**Type II Diabetes, Liver cancer, Chronic liver diseases: Obesity-attributable cumulative incidence, 95% Confidence Limits**				
Males					
20-24 years	4,992 [± 86]	29,469 [± 214]	55,101 [± 295]	82,938 [± 367]	109,865 [± 425]
25-29 years	3,981 [± 89]	22,292 [± 213]	38,963 [± 284]	55,992 [± 342]	73,401 [± 394]
30-34 years	5,345 [± 116]	30,579 [± 276]	52,831 [± 363]	72,961 [± 429]	92,884 [± 485]
35-39 years	12,977 [± 162]	78,153 [± 401]	146,169 [± 544]	214,359 [± 656]	283,949 [± 754]
40-44 years	13,691 [± 152]	81,244 [± 386]	148,755 [± 536]	220,328 [± 655]	291,957 [± 754]
45-49 years	11,761 [± 132]	78,216 [± 349]	148,709 [± 501]	220,586 [± 629]	297,794 [± 738]
50-54 years	7,762 [± 104]	57,088 [± 289]	120,051 [± 430]	187,872 [± 559]	256,709 [± 674]
55-59 years	4,358 [± 79]	31,313 [± 216]	67,633 [± 326]	112,738 [± 433]	158,840 [± 535]
Total males	**64,866 [± 336]**	**408,355 [± 854]**	**778,212 [± 1,194]**	**1,167,775 [± 1,480]**	**1,565,398 [± 1,730]**
Females					
20-24 years	3,734 [± 75]	20,904 [± 178]	37,801 [± 242]	55,133 [± 307]	71,032 [± 363]
25-29 years	2,404 [± 65]	12,522 [± 148]	20,594 [± 190]	28,541 [± 226]	36,568 [± 265]
30-34 years	2,872 [± 75]	16,402 [± 178]	27,060 [± 229]	35,573 [± 264]	43,895 [± 294]
35-39 years	7,069 [± 108]	42,985 [± 265]	78,500 [± 354]	109,163 [± 414]	135,942 [± 459]
40-44 years	7,132 [± 105]	42,230 [± 262]	77,103 [± 355]	111,685 [± 425]	141,559 [± 476]
45-49 years	4,534 [± 82]	31,266 [± 218]	59,312 [± 307]	87,243 [± 376]	114,897 [± 431]

50-54 years	2,503 [± 59]	18,279 [± 162]	39,306 [± 244]	61,492 [± 312]	83,456 [± 368]
55-59 years	1,702 [± 48]	11,732 [± 128]	26,826 [± 197]	47,993 [± 270]	71,294 [± 336]
Total females	**31,950 [± 225]**	**196,319 [± 561]**	**366,503 [± 769]**	**536,822 [± 937]**	**698,642 [± 1,077]**
**TOTAL**	**96,816 [± 404]**	**604,674 [± 1,021]**	**1,144,715 [± 1,420]**	**1,704,597 [± 1,752]**	**2,264,041 [± 2,038]**
**Type II Diabetes: Obesity-attributable cumulative incidence, 95% Confidence Limits**					
Males					
20-24 years	4,423 [± 77]	26,181 [± 192]	48,968 [± 265]	73,749 [± 329]	97,752 [± 381]
25-29 years	3,182 [± 74]	17,827 [± 176]	31,167 [± 235]	44,819 [± 282]	58,783 [± 324]
30-34 years	4,127 [± 94]	23,636 [± 221]	40,764 [± 291]	56,261 [± 343]	71,642 [± 387]
35-39 years	10,466 [± 139]	63,041 [± 346]	117,979 [± 470]	173,230 [± 567]	229,819 [± 653]
40-44 years	11,732 [± 136]	69,865 [± 347]	128,027 [± 483]	189,738 [± 590]	251,550 [± 680]
45-49 years	10,769 [± 124]	71,794 [± 328]	136,557 [± 472]	202,629 [± 592]	273,601 [± 696]
50-54 years	7,237 [± 99]	53,288 [± 274]	112,042 [± 408]	175,409 [± 530]	239,633 [± 640]
55-59 years	3,895 [± 72]	27,958 [± 198]	60,311 [± 298]	100,503 [± 397]	141,549 [± 489]
Total males	**55,831 [± 297]**	**353,589 [± 761]**	**675,815 [± 1,068]**	**1,016,338 [± 1,329]**	**1,364,328 [± 1,557]**
Females					
20-24 years	3,325 [± 67]	18,660 [± 160]	33,760 [± 217]	49,285 [± 275]	63,472 [± 324]
25-29 years	1,984 [± 55]	10,330 [± 125]	17,017 [± 161]	23,619 [± 190]	30,327 [± 222]
30-34 years	2,347 [± 63]	13,297 [± 149]	21,973 [± 191]	28,890 [± 219]	35,749 [± 244]
35-39 years	5,896 [± 95]	35,802 [± 234]	65,403 [± 312]	90,948 [± 365]	113,294 [± 404]
40-44 years	6,130 [± 95]	36,344 [± 236]	66,359 [± 320]	96,158 [± 383]	121,913 [± 428]

45-49 years	4,106 [± 76]	28,337 [± 203]	53,693 [± 286]	78,966 [± 349]	103,981 [± 400]
50-54 years	2,329 [± 56]	17,030 [± 154]	36,629 [± 231]	57,290 [± 296]	77,777 [± 349]
55-59 years	1,558 [± 45]	10,798 [± 120]	24,714 [± 185]	44,254 [± 253]	65,752 [± 315]
Total females	**27,676 [± 202]**	**170,597 [± 503]**	**319,548 [± 692]**	**469,409 [± 844]**	**612,265 [± 971]**
TOTAL	**83,507 [± 359]**	**524,186 [± 912]**	**995,363 [± 1,273]**	**1,485,748 [± 1,574]**	**1,976,593 [± 1,834]**
**Liver cancer: Obesity-attributable cumulative incidence, 95% Confidence Limits**					
Males					
20-24 years	0 [± 1]	2 [± 4]	3 [± 5]	3 [± 7]	5 [± 8]
25-29 years	0 [± 1]	0 [± 5]	1 [± 7]	2 [± 8]	4 [± 9]
30-34 years	1 [± 3]	3 [± 8]	8 [± 11]	10 [± 13]	11 [± 15]
35-39 years	0 [± 4]	7 [± 10]	14 [± 14]	25 [± 17]	32 [± 19]
40-44 years	5 [± 7]	33 [± 18]	59 [± 25]	79 [± 30]	104 [± 34]
45-49 years	5 [± 7]	35 [± 19]	72 [± 27]	99 [± 33]	137 [± 38]
50-54 years	18 [± 12]	136 [± 34]	272 [± 50]	418 [± 63]	563 [± 76]
55-59 years	16 [± 13]	129 [± 36]	291 [± 54]	475 [± 70]	651 [± 87]
Total males	**47 [± 21]**	**346 [± 58]**	**720 [± 85]**	**1,111 [± 107]**	**1,506 [± 129]**
Females					
20-24 years	0 [± 1]	1 [± 3]	1 [± 5]	3 [± 7]	4 [± 8]
25-29 years	0 [± 0]	0 [± 0]	0 [± 0]	0 [± 0]	0 [± 0]
30-34 years	0 [± 0]	0 [± 0]	0 [± 0]	0 [± 0]	0 [± 0]
35-39 years	0 [± 0]	0 [± 0]	0 [± 0]	0 [± 0]	0 [± 0]
40-44 years	3 [± 5]	13 [± 12]	24 [± 16]	37 [± 20]	48 [± 22]
45-49 years	4 [± 5]	31 [± 16]	55 [± 23]	77 [± 29]	106 [± 33]

50-54 years	3 [± 6]	24 [± 16]	60 [± 25]	98 [± 32]	134 [± 38]
55-59 years	6 [± 7]	48 [± 19]	121 [± 30]	210 [± 41]	304 [± 52]
Total females	**17 [± 12]**	**117 [± 32]**	**262 [± 49]**	**425 [± 63]**	**595 [± 76]**
**TOTAL**	**63 [± 25]**	**463 [± 66]**	**982 [± 98]**	**1,535 [± 125]**	**2,101 [± 150]**
	**Chronic liver diseases: Obesity-attributable cumulative incidence, 95% Confidence Limits**				
Males					
20-24 years	569 [± 37]	3,287 [± 94]	6,130 [± 130]	9,187 [± 162]	12,108 [± 188]
25-29 years	799 [± 50]	4,465 [± 120]	7,795 [± 161]	11,171 [± 194]	14,614 [± 223]
30-34 years	1,217 [± 69]	6,940 [± 165]	12,059 [± 218]	16,690 [± 257]	21,230 [± 291]
35-39 years	2,511 [± 83]	15,105 [± 203]	28,176 [± 275]	41,104 [± 329]	54,098 [± 376]
40-44 years	1,954 [± 67]	11,347 [± 168]	20,669 [± 232]	30,511 [± 282]	40,303 [± 323]
45-49 years	986 [± 45]	6,387 [± 117]	12,080 [± 166]	17,859 [± 208]	24,056 [± 242]
50-54 years	507 [± 31]	3,664 [± 85]	7,736 [± 126]	12,045 [± 163]	16,514 [± 196]
55-59 years	447 [± 30]	3,226 [± 80]	7,032 [± 120]	11,760 [± 160]	16,640 [± 197]
Total males	**8,988 [± 154]**	**54,420 [± 383]**	**101,677 [± 526]**	**150,326 [± 643]**	**199,564 [± 743]**
Females					
20-24 years	409 [± 32]	2,243 [± 77]	4,040 [± 106]	5,845 [± 136]	7,556 [± 163]
25-29 years	420 [± 34]	2,192 [± 79]	3,578 [± 102]	4,922 [± 122]	6,241 [± 145]
30-34 years	525 [± 41]	3,105 [± 98]	5,087 [± 127]	6,682 [± 147]	8,146 [± 165]
35-39 years	1,173 [± 51]	7,182 [± 124]	13,097 [± 167]	18,216 [± 196]	22,648 [± 217]
40-44 years	998 [± 45]	5,873 [± 113]	10,720 [± 153]	15,490 [± 184]	19,598 [± 207]

45-49 years	424 [± 29]	2,898 [± 77]	5,564 [± 110]	8,200 [± 135]	10,810 [± 155]
50-54 years	171 [± 17]	1,225 [± 48]	2,616 [± 73]	4,105 [± 94]	5,545 [± 111]
55-59 years	138 [± 15]	887 [± 40]	1,990 [± 61]	3,529 [± 83]	5,239 [± 103]
Total females	**4,258 [± 99]**	**25,605 [± 245]**	**46,692 [± 332]**	**66,988 [± 402]**	**85,783 [± 460]**
**TOTAL**	**13,246 [± 184]**	**80,025 [± 455]**	**148,369 [± 623]**	**217,314 [± 758]**	**285,346 [± 874]**
**Cumulative obesity-attributable healthcare costs (USD): T2DM, Liver cancer, Chronic liver diseases (95% Confidence Limits)**					
Males					
20-24 years	118,497,996	706,100,397	1,321,299,472	1,988,380,183	2,637,945,467
	[± 1,366,614]	[± 3,408,324]	[± 4,718,306]	[± 5,907,677]	[± 6,890,933]
25-29 years	227,331,768	1,400,465,001	2,508,005,518	3,634,963,200	4,794,214,104
	[± 2,122,368]	[± 5,037,549]	[± 6,736,468]	[± 8,117,757]	[± 9,370,890]
30-34 years	259,220,613	1,886,957,200	3,687,724,682	5,355,440,194	7,009,187,968
	[± 3,111,533]	[± 7,206,775]	[± 9,398,309]	[± 11,068,410]	[± 12,485,417]
35-39 years	348,664,016	2,402,466,201	5,084,437,292	7,928,662,416	10,618,673,881
	[± 3,818,654]	[± 9,115,704]	[± 11,942,168]	[± 13,980,267]	[± 15,669,003]
40-44 years	475,640,284	3,155,955,070	6,321,740,708	10,193,470,533	14,236,325,214
	[± 4,008,793]	[± 9,903,908]	[± 13,337,023]	[± 15,817,063]	[± 17,771,614]
45-49 years	412,418,871	3,272,131,868	6,869,177,670	10,983,946,730	15,899,689,645
	[± 3,703,639]	[± 9,561,680]	[± 13,316,332]	[± 16,234,477]	[± 18,547,168]
50-54 years	264,450,629	2,403,671,241	5,977,508,967	10,356,852,007	15,272,613,821

	[± 3,192,907]	[± 8,489,961]	[± 12,242,487]	[± 15,438,662]	[± 18,129,359]
55-59 years	167,143,603	1,533,239,717	4,148,321,817	8,289,167,816	13,217,241,869
	[± 2,725,333]	[± 7,328,128]	[± 10,789,963]	[± 14,017,484]	[± 16,979,791]
Total males	**2,273,367,779**	**16,760,986,696**	**35,918,216,125**	**58,730,883,080**	**83,685,891,970**
	**[± 8,834,325]**	**[± 22,075,354]**	**[± 30,332,964]**	**[± 36,977,507]**	**[± 42,585,402]**
Females					
20-24 years	97,359,426	541,285,353	977,206,148	1,423,515,391	1,839,214,475
	[± 1,281,033]	[± 3,085,583]	[± 4,252,021]	[± 5,491,202]	[± 6,596,138]
25-29 years	165,738,494	966,381,537	1,634,690,295	2,284,337,548	2,943,771,342
	[± 1,759,868]	[± 3,999,559]	[± 5,182,262]	[± 6,189,184]	[± 7,357,783]
30-34 years	150,811,138	1,138,482,552	2,170,863,724	3,020,955,022	3,841,689,537
	[± 2,190,588]	[± 5,124,399]	[± 6,552,651]	[± 7,541,041]	[± 8,443,682]
35-39 years	175,697,197	1,271,976,178	2,771,179,247	4,244,256,703	5,468,904,320
	[± 2,557,741]	[± 6,133,167]	[± 8,040,348]	[± 9,273,873]	[± 10,193,343]
40-44 years	251,208,545	1,665,158,617	3,383,838,551	5,495,638,110	7,499,060,200
	[± 2,735,695]	[± 6,761,779]	[± 9,074,027]	[± 10,713,032]	[± 11,863,247]
45-49 years	210,614,286	1,712,464,800	3,619,385,910	5,828,564,372	8,425,523,960
	[± 2,520,331]	[± 6,628,882]	[± 9,273,991]	[± 11,254,187]	[± 12,770,117]
50-54 years	114,634,108	1,114,667,555	2,949,235,289	5,207,192,128	7,763,305,187
	[± 2,101,979]	[± 5,738,668]	[± 8,516,591]	[± 10,821,564]	[± 12,665,743]
55-59 years	67,242,594	615,283,914	1,822,435,700	3,933,522,122	6,489,147,548
	[± 1,821,325]	[± 4,809,260]	[± 7,264,386]	[± 9,709,776]	[± 11,882,430]
Total females	**1,233,305,788**	**9,025,700,506**	**19,328,834,864**	**31,437,981,395**	**44,270,616,570**

	**[± 6,136,003]**	**[± 15,333,543]**	**[± 21,122,700]**	**[± 25,770,974]**	**[± 29,635,649]**
**TOTAL**	**3,506,673,566**	**25,786,687,202**	**55,247,050,989**	**90,168,864,475**	**127,956,508,540**
	**[± 10,756,200]**	**[± 26,878,222]**	**[± 36,962,916]**	**[± 45,071,933]**	**[± 51,882,446]**
**Cumulative obesity-attributable healthcare costs (USD): T2DM (95% Confidence Limits)**					
Males					
20-24 years	93,290,658	557,256,093	1,044,256,855	1,573,677,914	2,089,659,033
	[± 637,346]	[± 1,575,884]	[± 2,175,773]	[± 2,710,855]	[± 3,147,532]
25-29 years	156,449,081	1,000,513,002	1,808,727,782	2,633,886,317	3,486,358,432
	[± 1,034,326]	[± 2,380,343]	[± 3,136,683]	[± 3,754,021]	[± 4,308,561]
30-34 years	152,850,250	1,167,656,646	2,375,066,630	3,508,195,793	4,636,563,231
	[± 1,520,903]	[± 3,442,844]	[± 4,377,092]	[± 5,073,077]	[± 5,666,948]
35-39 years	187,323,411	1,328,761,995	2,929,415,653	4,723,509,047	6,435,445,018
	[± 1,845,240]	[± 4,373,098]	[± 5,643,638]	[± 6,493,904]	[± 7,190,988]
40-44 years	273,421,882	1,803,750,925	3,637,115,698	5,975,685,044	8,511,252,264
	[± 1,927,908]	[± 4,765,639]	[± 6,399,610]	[± 7,524,362]	[± 8,364,453]
45-49 years	269,414,283	2,084,091,478	4,312,411,316	6,866,723,113	9,991,115,133
	[± 1,804,808]	[± 4,666,966]	[± 6,513,974]	[± 7,939,970]	[± 9,031,566]
50-54 years	197,062,485	1,724,748,449	4,151,655,693	7,053,918,232	10,295,460,446
	[± 1,588,738]	[± 4,224,398]	[± 6,102,876]	[± 7,718,142]	[± 9,074,417]
55-59 years	130,007,236	1,171,670,791	3,074,623,688	5,955,388,302	9,307,768,382
	[± 1,377,541]	[± 3,689,642]	[± 5,428,026]	[± 7,061,196]	[± 8,571,594]

Total males	**1,459,819,285 [± 4,311,608]**	**10,838,449,380 [± 10,727,418]**	**23,333,273,316 [± 14,685,972]**	**38,290,983,763 [± 17,844,821]**	**54,753,621,938 [± 20,485,258]**
Females					
20-24 years	76,227,420 [± 579,999]	427,689,605 [± 1,376,322]	774,363,480 [± 1,872,028]	1,130,196,151 [± 2,374,607]	1,462,014,758 [± 2,816,513]
25-29 years	116,256,568 [± 857,380]	701,388,727 [± 1,883,642]	1,202,195,545 [± 2,395,559]	1,692,318,141 [± 2,819,839]	2,190,963,677 [± 3,294,933]
30-34 years	95,181,500 [± 1,103,687]	741,957,383 [± 2,516,134]	1,464,762,042 [± 3,132,775]	2,074,306,953 [± 3,538,949]	2,667,590,509 [± 3,902,389]
35-39 years	102,954,194 [± 1,289,406]	763,259,632 [± 3,072,830]	1,715,483,065 [± 3,964,314]	2,703,321,171 [± 4,490,854]	3,538,323,334 [± 4,867,623]
40-44 years	153,829,747 [± 1,379,917]	1,013,651,992 [± 3,404,520]	2,068,569,730 [± 4,549,804]	3,408,822,874 [± 5,316,444]	4,733,021,602 [± 5,816,599]
45-49 years	138,208,061 [± 1,284,709]	1,106,023,869 [± 3,368,171]	2,313,756,817 [± 4,704,538]	3,714,089,743 [± 5,691,545]	5,396,513,592 [± 6,411,175]
50-54 years	82,616,545 [± 1,088,048]	777,931,706 [± 2,962,711]	2,009,811,063 [± 4,385,219]	3,495,397,707 [± 5,562,899]	5,171,645,247 [± 6,494,479]
55-59 years	52,381,069 [± 960,416]	465,233,844 [± 2,524,596]	1,327,968,860 [± 3,795,104]	2,785,986,210 [± 5,056,253]	4,515,500,277 [± 6,175,858]
Total females	**817,655,103 [± 3,100,498]**	**5,997,136,758 [± 7,700,790]**	**12,876,910,602 [± 10,547,019]**	**21,004,438,952 [± 12,784,834]**	**29,675,572,997 [± 14,593,395]**
**TOTAL**	**2,277,474,389 [± 5,310,655]**	**16,835,586,138 [± 13,205,289]**	**36,210,183,917 [± 18,080,857]**	**59,295,422,715 [± 21,951,985]**	**84,429,194,935 [± 25,151,799]**

	Cumulative obesity-attributable healthcare costs (USD): Liver cancer (95% Confidence Limits)				
Males					
20-24 years	3,882 [± 118,335]	32,487 [± 313,077]	51,017 [± 419,314]	92,917 [± 525,052]	119,878 [± 623,879]
25-29 years	42,178 [± 157,765]	164,186 [± 372,339]	228,017 [± 519,475]	289,813 [± 620,073]	425,943 [± 716,355]
30-34 years	28,119 [± 207,647]	300,147 [± 480,822]	644,458 [± 636,571]	806,020 [± 770,821]	960,728 [± 866,598]
35-39 years	44,978 [± 213,386]	491,941 [± 537,604]	1,188,539 [± 724,441]	1,914,818 [± 866,793]	2,343,807 [± 996,286]
40-44 years	158,182 [± 234,874]	869,556 [± 557,440]	1,612,845 [± 760,439]	2,592,471 [± 918,867]	3,539,188 [± 1,049,328]
45-49 years	107,364 [± 196,353]	788,570 [± 526,711]	1,639,499 [± 734,566]	2,410,164 [± 901,433]	3,692,333 [± 1,045,822]
50-54 years	270,335 [± 232,773]	2,189,740 [± 624,190]	4,607,692 [± 914,437]	7,126,591 [± 1,154,725]	9,691,968 [± 1,368,184]
55-59 years	285,434 [± 224,275]	2,186,593 [± 614,979]	4,866,833 [± 935,471]	8,151,942 [± 1,219,377]	11,442,804 [± 1,488,636]
Total males	940,472 [± 570,743]	7,023,220 [± 1,453,560]	14,838,899 [± 2,050,578]	23,384,736 [± 2,546,141]	32,216,649 [± 2,988,104]
Females					
20-24 years	15,157 [± 106,917]	91,819 [± 264,726]	122,565 [± 360,698]	245,477 [± 469,064]	339,710 [± 565,652]
25-29 years	29,783 [± 106,120]	124,547 [± 260,146]	184,292 [± 359,501]	235,406 [± 435,957]	358,569 [± 525,397]
30-34 years	4,670 [± 104,740]	109,944 [± 254,444]	191,898 [± 343,100]	241,350 [± 418,862]	289,871 [± 483,226]
35-39 years	-4,803 [± 104,271]	-5,264 [± 248,010]	110,501 [± 331,269]	193,439 [± 397,094]	243,540 [± 457,575]
40-44 years	68,350 [± 81,009]	328,670 [± 208,236]	649,962 [± 300,601]	934,492 [± 369,436]	1,221,849 [± 411,013]
45-49 years	118,869 [± 110,985]	934,409 [± 317,494]	1,603,858 [± 460,020]	2,202,212 [± 568,855]	3,054,224 [± 657,697]
50-54 years	130,334 [± 122,775]	888,440 [± 333,630]	2,037,840 [± 505,067]	3,194,807 [± 650,656]	4,216,484 [± 774,258]
55-59 years	172,430 [± 137,794]	1,304,096 [± 372,896]	3,158,893 [± 584,522]	5,626,243 [± 806,651]	8,410,633 [± 1,002,952]
Total females	**534,790 [± 312,212]**	**3,776,661 [± 811,500]**	**8,059,809 [± 1,177,090]**	**12,873,426 [± 1,509,082]**	**18,134,879 [± 1,801,602]**

**TOTAL**	**1,475,262 [± 650,556]**	**10,799,881 [± 1,664,743]**	**22,898,708 [± 2,364,405]**	**36,258,162 [± 2,959,757]**	**50,351,528 [± 3,489,203]**
**Cumulative obesity-attributable healthcare costs (USD): Chronic liver diseases (95% Confidence Limits)**					
Males					
20-24 years	25,203,456	148,811,817	276,991,600	414,609,351	548,166,557
	[± 1,203,088]	[± 3,005,868]	[± 4,165,646]	[± 5,222,666]	[± 6,098,260]
25-29 years	70,840,509	399,787,813	699,049,719	1,000,787,070	1,307,429,730
	[± 1,846,544]	[± 4,424,052]	[± 5,938,971]	[± 7,170,831]	[± 8,290,761]
30-34 years	106,342,244	719,000,407	1,312,013,595	1,846,438,382	2,371,664,009
	[± 2,706,543]	[± 6,312,942]	[± 8,292,410]	[± 9,807,110]	[± 11,091,453]
35-39 years	161,295,627	1,073,212,265	2,153,833,099	3,203,238,551	4,180,885,056
	[± 3,336,417]	[± 7,980,166]	[± 10,499,520]	[± 12,350,132]	[± 13,885,776]
40-44 years	202,060,220	1,351,334,589	2,683,012,166	4,215,193,019	5,721,533,762
	[± 3,506,911]	[± 8,664,026]	[± 11,676,597]	[± 13,882,332]	[± 15,644,970]
45-49 years	142,897,223	1,187,251,820	2,555,126,855	4,114,813,453	5,904,882,179
	[± 3,228,166]	[± 8,328,730]	[± 11,591,085]	[± 14,131,615]	[± 16,165,844]
50-54 years	67,117,809	676,733,052	1,821,245,582	3,295,807,183	4,967,461,407
	[± 2,759,779]	[± 7,337,866]	[± 10,573,420]	[± 13,321,005]	[± 15,635,110]
55-59 years	36,850,933	359,382,333	1,068,831,296	2,325,627,572	3,898,030,683
	[± 2,340,838]	[± 6,301,572]	[± 9,278,186]	[± 12,047,509]	[± 14,581,668]
Total males	**812,608,022**	**5,915,514,096**	**12,570,103,910**	**20,416,514,582**	**28,900,053,383**

	**[± 7,689,576]**	**[± 19,238,787]**	**[± 26,461,407]**	**[± 32,286,461]**	**[± 37,214,807]**
Females					
20-24 years	21,116,849<br>[± 1,137,196]	113,503,929<br>[± 2,748,906]	202,720,103<br>[± 3,800,669]	293,073,762<br>[± 4,928,947]	376,860,008<br>[± 5,937,704]
25-29 years	49,452,142<br>[± 1,533,224]	264,868,264<br>[± 3,518,620]	432,310,459<br>[± 4,581,255]	591,784,000<br>[± 5,492,217]	752,449,096<br>[± 6,557,770]
30-34 years	55,624,968<br>[± 1,889,333]	396,415,224<br>[± 4,456,882]	705,909,783<br>[± 5,745,018]	946,406,718<br>[± 6,645,878]	1,173,809,157<br>[± 7,472,190]
35-39 years	72,747,806<br>[± 2,206,490]	508,721,810<br>[± 5,302,070]	1,055,585,681<br>[± 6,987,251]	1,540,742,093<br>[± 8,104,274]	1,930,337,446<br>[± 8,944,334]
40-44 years	97,310,448<br>[± 2,360,782]	651,177,955<br>[± 5,838,454]	1,314,618,859<br>[± 7,845,182]	2,085,880,744<br>[± 9,293,438]	2,764,816,749<br>[± 10,331,257]
45-49 years	72,287,356<br>[± 2,165,474]	605,506,522<br>[± 5,700,588]	1,304,025,235<br>[± 7,978,885]	2,112,272,417<br>[± 9,692,236]	3,025,956,144<br>[± 11,024,525]
50-54 years	31,887,230<br>[± 1,794,267]	335,847,408<br>[± 4,903,402]	937,386,386<br>[± 7,283,343]	1,708,599,614<br>[± 9,259,430]	2,587,443,456<br>[± 10,846,350]
55-59 years	14,689,095<br>[± 1,541,375]	148,745,975<br>[± 4,076,315]	491,307,947<br>[± 6,166,590]	1,141,909,669<br>[± 8,250,052]	1,965,236,639<br>[± 10,101,734]
Total females	**415,115,894**<br>**[± 5,285,827]**	**3,024,787,087**<br>**[± 13,234,683]**	**6,443,864,454**<br>**[± 18,263,168]**	**10,420,669,017**<br>**[± 22,325,182]**	**14,576,908,694**<br>**[± 25,730,502]**
**TOTAL**	**1,227,723,916**<br>**[± 9,331,107]**	**8,940,301,183**<br>**[± 23,351,398]**	**19,013,968,364**<br>**[± 32,151,973]**	**30,837,183,599**<br>**[± 39,253,399]**	**43,476,962,077**<br>**[± 45,243,791]**
