# Supplementary material for: The future health and economic burden of obesity-attributable type 2 diabetes and liver disease among the working-age population in Saudi Arabia
Source: PLoS One. 2022 Jul 14;17(7):e0271108. doi: 10.1371/journal.pone.0271108 (PMC9282435; doi:10.1371/journal.pone.0271108)
Supplement: S3 Table — Projected obesity-attributable annual incidence (a) and prevalence (b) of T2DM, chronic liver diseases and liver cancer, by sex and working age group (2020 to 2040). (PDF) [file pone.0271108.s003.pdf]

S3 Table: Projected obesity-attributable annual incidence (a) and prevalence (b) of T2DM, chronic liver diseases and liver cancer, by sex and working age group (2020 to 2040)

Indicator, by sex and age	2020	2025	2030	2035	2040
**(a)**					
**Type II Diabetes, Liver cancer, Chronic liver disease: Obesity-attributable annual incidence, 95% Confidence Limits**					
Males					
20-24 years	4,992 [± 86]	4,818 [± 87]	5,327 [± 92]	5,571 [± 99]	5,185 [± 93]
25-29 years	3,981 [± 89]	3,535 [± 85]	3,273 [± 85]	3,520 [± 86]	3,440 [± 87]
30-34 years	5,345 [± 116]	4,857 [± 111]	4,177 [± 104]	4,012 [± 103]	3,993 [± 99]
35-39 years	12,977 [± 162]	13,184 [± 165]	13,905 [± 166]	13,570 [± 163]	14,432 [± 169]
40-44 years	13,691 [± 152]	13,267 [± 163]	13,842 [± 168]	14,507 [± 169]	14,149 [± 166]
45-49 years	11,761 [± 132]	14,168 [± 152]	14,081 [± 166]	14,701 [± 172]	15,854 [± 174]
50-54 years	7,762 [± 104]	11,221 [± 130]	13,297 [± 150]	13,334 [± 163]	14,224 [± 171]
55-59 years	4,358 [± 79]	6,111 [± 97]	8,017 [± 116]	9,490 [± 134]	9,041 [± 143]
Total males	**64,866 [± 336]**	**71,159 [± 361]**	**75,919 [± 381]**	**78,704 [± 396]**	**80,319 [± 403]**
Females					
20-24 years	3,734 [± 75]	3,307 [± 71]	3,452 [± 75]	3,398 [± 89]	2,990 [± 83]
25-29 years	2,404 [± 65]	1,824 [± 56]	1,568 [± 53]	1,612 [± 55]	1,582 [± 66]
30-34 years	2,872 [± 75]	2,547 [± 71]	1,906 [± 61]	1,626 [± 58]	1,685 [± 59]
35-39 years	7,069 [± 108]	7,258 [± 107]	6,864 [± 103]	5,661 [± 91]	5,310 [± 88]
40-44 years	7,132 [± 105]	6,886 [± 107]	6,972 [± 106]	6,740 [± 103]	5,494 [± 91]
45-49 years	4,534 [± 82]	5,676 [± 95]	5,570 [± 97]	5,613 [± 96]	5,267 [± 92]
50-54 years	2,503 [± 59]	3,617 [± 73]	4,486 [± 86]	4,331 [± 87]	4,363 [± 86]
55-59 years	1,702 [± 48]	2,341 [± 58]	3,571 [± 74]	4,630 [± 87]	4,620 [± 90]
Total females	**31,950 [± 225]**	**33,457 [± 232]**	**34,391 [± 237]**	**33,611 [± 240]**	**31,311 [± 234]**
**TOTAL**	**96,816 [± 404]**	**104,616 [± 429]**	**110,309 [± 449]**	**112,314 [± 463]**	**111,630 [± 466]**
**Type II Diabetes: Obesity-attributable annual incidence, 95% Confidence Limits**					
Males					
20-24 years	4,423 [± 77]	4,291 [± 78]	4,717 [± 82]	4,958 [± 89]	4,631 [± 83]
25-29 years	3,182 [± 74]	2,808 [± 70]	2,644 [± 70]	2,825 [± 71]	2,738 [± 71]

30-34 years	4,127 [± 94]	3,751 [± 88]	3,208 [± 83]	3,123 [± 82]	3,102 [± 79]
35-39 years	10,466 [± 139]	10,662 [± 142]	11,275 [± 143]	10,979 [± 142]	11,765 [± 147]
40-44 years	11,732 [± 136]	11,403 [± 146]	11,921 [± 151]	12,488 [± 152]	12,272 [± 151]
45-49 years	10,769 [± 124]	12,985 [± 144]	12,928 [± 156]	13,512 [± 162]	14,556 [± 165]
50-54 years	7,237 [± 99]	10,488 [± 123]	12,400 [± 143]	12,472 [± 155]	13,262 [± 163]
55-59 years	3,895 [± 72]	5,471 [± 89]	7,148 [± 106]	8,463 [± 123]	8,063 [± 131]
Total males	**55,831 [± 297]**	**61,858 [± 323]**	**66,242 [± 343]**	**68,822 [± 358]**	**70,388 [± 365]**
Females					
20-24 years	3,325 [± 67]	2,945 [± 64]	3,101 [± 67]	3,031 [± 79]	2,662 [± 74]
25-29 years	1,984 [± 55]	1,494 [± 47]	1,313 [± 45]	1,335 [± 46]	1,324 [± 54]
30-34 years	2,347 [± 63]	2,075 [± 59]	1,544 [± 50]	1,337 [± 48]	1,387 [± 49]
35-39 years	5,896 [± 95]	6,044 [± 95]	5,745 [± 91]	4,708 [± 80]	4,426 [± 78]
40-44 years	6,130 [± 95]	5,917 [± 97]	5,985 [± 95]	5,802 [± 92]	4,737 [± 82]
45-49 years	4,106 [± 76]	5,139 [± 88]	5,020 [± 90]	5,068 [± 89]	4,792 [± 85]
50-54 years	2,329 [± 56]	3,387 [± 70]	4,176 [± 81]	4,037 [± 83]	4,063 [± 81]
55-59 years	1,558 [± 45]	2,152 [± 54]	3,292 [± 70]	4,264 [± 82]	4,267 [± 85]
Total females	**27,676 [± 202]**	**29,153 [± 209]**	**30,177 [± 214]**	**29,583 [± 217]**	**27,657 [± 211]**
**TOTAL**	**83,507 [± 359]**	**91,012 [± 385]**	**96,419 [± 405]**	**98,405 [± 419]**	**98,045 [± 422]**
**Liver cancer: Obesity-attributable annual incidence, 95% Confidence Limits**					
Males					
20-24 years	0 [± 1]	0 [± 1]	0 [± 1]	0 [± 2]	0 [± 1]
25-29 years	0 [± 1]	0 [± 2]	0 [± 2]	1 [± 2]	0 [± 2]
30-34 years	1 [± 3]	0 [± 3]	0 [± 3]	0 [± 3]	0 [± 3]
35-39 years	0 [± 4]	1 [± 4]	1 [± 2]	0 [± 5]	1 [± 4]
40-44 years	5 [± 7]	5 [± 7]	6 [± 8]	5 [± 8]	3 [± 7]
45-49 years	5 [± 7]	6 [± 8]	9 [± 9]	3 [± 8]	8 [± 9]
50-54 years	18 [± 12]	26 [± 15]	31 [± 18]	33 [± 18]	30 [± 19]
55-59 years	16 [± 13]	27 [± 16]	38 [± 19]	36 [± 21]	34 [± 24]
Total males	**47 [± 21]**	**65 [± 25]**	**85 [± 29]**	**78 [± 31]**	**77 [± 33]**
Females					
20-24 years	0 [± 1]	0 [± 2]	0 [± 1]	0 [± 2]	0 [± 1]

25-29 years	0 [± 0]	0 [± 0]	0 [± 0]	0 [± 0]	0 [± 0]
30-34 years	0 [± 0]	0 [± 0]	0 [± 0]	0 [± 0]	0 [± 0]
35-39 years	0 [± 0]	0 [± 0]	0 [± 0]	0 [± 0]	0 [± 0]
40-44 years	3 [± 5]	3 [± 5]	2 [± 5]	4 [± 5]	0 [± 4]
45-49 years	4 [± 5]	4 [± 7]	3 [± 7]	6 [± 7]	4 [± 8]
50-54 years	3 [± 6]	5 [± 7]	7 [± 9]	8 [± 10]	6 [± 9]
55-59 years	6 [± 7]	10 [± 9]	17 [± 11]	21 [± 13]	18 [± 14]
Total females	**17 [± 12]**	**21 [± 14]**	**29 [± 16]**	**37 [± 19]**	**29 [± 19]**
**TOTAL**	**63 [± 25]**	**87 [± 29]**	**114 [± 33]**	**116 [± 36]**	**106 [± 38]**
**Chronic liver diseases: Obesity-attributable annual incidence, 95% Confidence Limits**					
Males					
20-24 years	569 [± 37]	527 [± 38]	610 [± 41]	612 [± 44]	554 [± 41]
25-29 years	799 [± 50]	727 [± 48]	629 [± 47]	694 [± 49]	702 [± 51]
30-34 years	1,217 [± 69]	1,105 [± 67]	968 [± 63]	888 [± 62]	891 [± 60]
35-39 years	2,511 [± 83]	2,520 [± 83]	2,629 [± 83]	2,590 [± 80]	2,666 [± 82]
40-44 years	1,954 [± 67]	1,859 [± 71]	1,915 [± 72]	2,014 [± 72]	1,874 [± 69]
45-49 years	986 [± 45]	1,177 [± 51]	1,144 [± 55]	1,186 [± 56]	1,290 [± 57]
50-54 years	507 [± 31]	707 [± 38]	865 [± 44]	828 [± 47]	932 [± 50]
55-59 years	447 [± 30]	613 [± 35]	831 [± 43]	991 [± 49]	944 [± 53]
Total males	**8,988 [± 154]**	**9,236 [± 159]**	**9,591 [± 163]**	**9,804 [± 166]**	**9,853 [± 167]**
Females					
20-24 years	409 [± 32]	361 [± 31]	351 [± 33]	366 [± 40]	328 [± 38]
25-29 years	420 [± 34]	330 [± 31]	255 [± 29]	277 [± 31]	258 [± 37]
30-34 years	525 [± 41]	472 [± 39]	362 [± 34]	289 [± 33]	298 [± 34]
35-39 years	1,173 [± 51]	1,213 [± 51]	1,119 [± 49]	953 [± 44]	884 [± 42]
40-44 years	998 [± 45]	967 [± 46]	985 [± 46]	935 [± 45]	756 [± 39]
45-49 years	424 [± 29]	534 [± 34]	547 [± 35]	539 [± 35]	471 [± 34]
50-54 years	171 [± 17]	225 [± 21]	303 [± 26]	287 [± 26]	294 [± 27]
55-59 years	138 [± 15]	179 [± 18]	262 [± 23]	345 [± 27]	336 [± 28]
Total females	**4,258 [± 99]**	**4,282 [± 100]**	**4,185 [± 100]**	**3,990 [± 101]**	**3,625 [± 100]**
**TOTAL**	**13,246 [± 184]**	**13,518 [± 188]**	**13,776 [± 191]**	**13,794 [± 194]**	**13,478 [± 194]**

**(b)**					
**Type II Diabetes, Liver cancer, Chronic liver diseases: Obesity-attributable annual incidence per 100,000, 95% Confidence Limits**					
Males					
20-24 years	37,320 [± 272]	36,610 [± 276]	40,189 [± 291]	42,528 [± 319]	39,585 [± 298]
25-29 years	65,791 [± 435]	68,676 [± 395]	66,236 [± 396]	69,790 [± 399]	69,199 [± 410]
30-34 years	68,438 [± 639]	101,336 [± 560]	100,737 [± 510]	94,819 [± 501]	96,790 [± 489]
35-39 years	87,344 [± 778]	120,412 [± 727]	156,032 [± 656]	152,875 [± 608]	148,751 [± 605]
40-44 years	123,757 [± 814]	145,365 [± 822]	182,555 [± 792]	220,809 [± 731]	216,195 [± 687]
45-49 years	115,642 [± 759]	180,114 [± 835]	202,926 [± 861]	241,411 [± 840]	287,768 [± 796]
50-54 years	80,409 [± 664]	159,369 [± 769]	229,729 [± 852]	254,010 [± 891]	297,509 [± 883]
55-59 years	52,223 [± 573]	108,928 [± 680]	190,340 [± 779]	269,832 [± 878]	287,560 [± 913]
Total males	**630,926 [± 1,813]**	**920,809 [± 1,871]**	**1,168,744 [± 1,906]**	**1,346,072 [± 1,920]**	**1,443,356 [± 1,893]**
Females					
20-24 years	30,554 [± 250]	27,008 [± 240]	28,290 [± 253]	27,978 [± 309]	24,990 [± 292]
25-29 years	48,532 [± 361]	44,774 [± 297]	39,229 [± 283]	40,537 [± 294]	39,886 [± 357]
30-34 years	41,429 [± 460]	62,818 [± 402]	55,747 [± 334]	48,492 [± 316]	49,882 [± 327]
35-39 years	46,221 [± 537]	69,331 [± 506]	89,063 [± 449]	77,260 [± 379]	68,118 [± 360]
40-44 years	67,791 [± 575]	80,103 [± 578]	103,470 [± 549]	121,824 [± 496]	103,902 [± 423]
45-49 years	59,214 [± 534]	96,897 [± 603]	108,524 [± 610]	132,571 [± 583]	149,296 [± 533]
50-54 years	34,132 [± 450]	77,062 [± 548]	119,405 [± 626]	130,357 [± 634]	154,708 [± 609]
55-59 years	21,028 [± 395]	44,307 [± 459]	92,650 [± 565]	138,749 [± 646]	149,507 [± 656]
Total females	348,901 [± 1,291]	502,300 [± 1,331]	636,378 [± 1,357]	717,769 [± 1,353]	740,289 [± 1,309]
**TOTAL**	**979,826 [± 2,225]**	**1,423,108 [± 2,296]**	**1,805,122 [± 2,340]**	**2,063,841 [± 2,349]**	**2,183,645 [± 2,301]**
**Type II Diabetes: Obesity-attributable annual incidence per 100,000, 95% Confidence Limits**					
Males					
20-24 years	34,496 [± 236]	33,891 [± 238]	37,229 [± 251]	39,431 [± 274]	36,752 [± 256]
25-29 years	57,850 [± 382]	61,676 [± 341]	59,602 [± 342]	62,842 [± 343]	62,368 [± 352]
30-34 years	56,519 [± 562]	86,859 [± 485]	88,032 [± 434]	83,042 [± 426]	84,871 [± 415]
35-39 years	69,266 [± 682]	98,272 [± 634]	130,996 [± 564]	130,214 [± 516]	126,798 [± 514]

40-44 years	101,102 [± 713]	118,164 [± 719]	150,700 [± 690]	185,643 [± 631]	183,686 [± 589]
45-49 years	99,620 [± 667]	152,624 [± 735]	170,601 [± 758]	204,483 [± 738]	246,305 [± 694]
50-54 years	72,867 [± 587]	140,976 [± 681]	200,041 [± 756]	219,476 [± 790]	257,675 [± 782]
55-59 years	48,072 [± 509]	99,013 [± 603]	169,729 [± 691]	237,314 [± 781]	251,052 [± 812]
Total males	**539,792 [± 1,594]**	**791,475 [± 1,642]**	**1,006,931 [± 1,669]**	**1,162,444 [± 1,678]**	**1,249,507 [± 1,651]**
Females					
20-24 years	28,186 [± 214]	25,036 [± 204]	26,264 [± 214]	25,959 [± 258]	23,248 [± 243]
25-29 years	42,988 [± 317]	40,527 [± 256]	35,656 [± 242]	36,943 [± 250]	36,322 [± 300]
30-34 years	35,195 [± 408]	54,772 [± 351]	49,653 [± 286]	43,348 [± 268]	44,739 [± 276]
35-39 years	38,069 [± 477]	58,332 [± 447]	76,594 [± 391]	67,540 [± 324]	59,703 [± 306]
40-44 years	56,881 [± 510]	66,893 [± 513]	87,387 [± 484]	104,474 [± 432]	90,241 [± 364]
45-49 years	51,105 [± 475]	82,735 [± 536]	92,088 [± 541]	113,202 [± 516]	128,895 [± 466]
50-54 years	30,549 [± 402]	67,649 [± 490]	103,579 [± 558]	112,310 [± 565]	133,725 [± 541]
55-59 years	19,369 [± 355]	40,033 [± 411]	82,201 [± 506]	121,697 [± 578]	130,300 [± 586]
Total females	**302,341 [± 1,146]**	**435,977 [± 1,179]**	**553,422 [± 1,197]**	**625,474 [± 1,189]**	**647,174 [± 1,143]**
**TOTAL**	**842,134 [± 1,964]**	**1,227,452 [± 2,021]**	**1,560,353 [± 2,054]**	**1,787,918 [± 2,057]**	**1,896,680 [± 2,008]**
**Liver cancer: Obesity-attributable annual incidence per 100,000, 95% Confidence Limits**					
Males					
20-24 years	0 [± 9]	0 [± 10]	1 [± 9]	0 [± 11]	0 [± 11]
25-29 years	3 [± 12]	2 [± 11]	0 [± 13]	2 [± 11]	2 [± 12]
30-34 years	2 [± 16]	5 [± 14]	5 [± 14]	0 [± 15]	3 [± 13]
35-39 years	3 [± 16]	7 [± 17]	12 [± 16]	9 [± 16]	6 [± 16]
40-44 years	12 [± 18]	10 [± 16]	13 [± 18]	17 [± 18]	10 [± 17]
45-49 years	8 [± 15]	12 [± 17]	13 [± 17]	10 [± 18]	23 [± 18]
50-54 years	20 [± 18]	31 [± 21]	41 [± 24]	44 [± 24]	40 [± 25]
55-59 years	22 [± 17]	33 [± 21]	46 [± 25]	52 [± 28]	45 [± 29]
Total males	**71 [± 43]**	**99 [± 47]**	**131 [± 50]**	**135 [± 52]**	**129 [± 53]**
Females					
20-24 years	1 [± 8]	1 [± 8]	1 [± 8]	2 [± 11]	1 [± 10]

25-29 years	2 [± 8]	1 [± 8]	1 [± 9]	1 [± 8]	2 [± 11]
30-34 years	0 [± 8]	2 [± 8]	1 [± 8]	1 [± 8]	1 [± 8]
35-39 years	0 [± 8]	0 [± 7]	2 [± 7]	1 [± 7]	1 [± 8]
40-44 years	5 [± 6]	4 [± 7]	4 [± 7]	5 [± 7]	3 [± 5]
45-49 years	9 [± 8]	13 [± 11]	10 [± 11]	10 [± 11]	11 [± 11]
50-54 years	10 [± 9]	11 [± 11]	17 [± 14]	18 [± 14]	15 [± 14]
55-59 years	13 [± 10]	19 [± 13]	34 [± 17]	41 [± 19]	39 [± 21]
Total females	**40 [± 24]**	**51 [± 26]**	**68 [± 30]**	**79 [± 33]**	**72 [± 33]**
**TOTAL**	**111 [± 49]**	**150 [± 53]**	**199 [± 58]**	**214 [± 61]**	**202 [± 63]**
**Chronic liver diseases: Obesity-attributable annual incidence per 100,000, 95% Confidence Limits**					
Males					
20-24 years	2,824 [± 135]	2,720 [± 138]	2,959 [± 147]	3,097 [± 162]	2,833 [± 153]
25-29 years	7,939 [± 207]	6,998 [± 199]	6,634 [± 200]	6,946 [± 204]	6,829 [± 211]
30-34 years	11,917 [± 303]	14,472 [± 280]	12,700 [± 267]	11,777 [± 264]	11,916 [± 259]
35-39 years	18,075 [± 374]	22,133 [± 356]	25,024 [± 335]	22,652 [± 321]	21,946 [± 320]
40-44 years	22,643 [± 393]	27,191 [± 397]	31,842 [± 387]	35,149 [± 370]	32,499 [± 355]
45-49 years	16,013 [± 362]	27,479 [± 396]	32,312 [± 408]	36,918 [± 401]	41,440 [± 389]
50-54 years	7,521 [± 309]	18,362 [± 358]	29,647 [± 394]	34,490 [± 410]	39,794 [± 408]
55-59 years	4,130 [± 262]	9,881 [± 313]	20,565 [± 358]	32,466 [± 401]	36,464 [± 416]
Total males	**91,063 [± 862]**	**129,235 [± 897]**	**161,682 [± 919]**	**183,493 [± 931]**	**193,720 [± 924]**
Females					
20-24 years	2,366 [± 127]	1,971 [± 126]	2,025 [± 135]	2,016 [± 170]	1,741 [± 162]
25-29 years	5,542 [± 172]	4,246 [± 150]	3,572 [± 147]	3,593 [± 155]	3,562 [± 193]
30-34 years	6,233 [± 212]	8,044 [± 197]	6,093 [± 172]	5,144 [± 167]	5,143 [± 175]
35-39 years	8,152 [± 247]	10,998 [± 237]	12,467 [± 222]	9,719 [± 195]	8,414 [± 189]
40-44 years	10,905 [± 265]	13,206 [± 267]	16,079 [± 258]	17,345 [± 243]	13,658 [± 214]
45-49 years	8,101 [± 243]	14,149 [± 276]	16,427 [± 280]	19,359 [± 271]	20,389 [± 257]
50-54 years	3,573 [± 201]	9,402 [± 246]	15,809 [± 282]	18,029 [± 287]	20,968 [± 279]
55-59 years	1,646 [± 173]	4,255 [± 203]	10,415 [± 251]	17,011 [± 287]	19,168 [± 293]
Total females	**46,519 [± 592]**	**66,271 [± 618]**	**82,888 [± 637]**	**92,217 [± 645]**	**93,043 [± 637]**
**TOTAL**	**137,581 [± 1,046]**	**195,507 [± 1,089]**	**244,570 [± 1,119]**	**275,710 [± 1,133]**	**286,763 [± 1,122]**
