# Supplementary material for: The future health and economic burden of obesity-attributable type 2 diabetes and liver disease among the working-age population in Saudi Arabia
Source: PLoS One. 2022 Jul 14;17(7):e0271108. doi: 10.1371/journal.pone.0271108 (PMC9282435; doi:10.1371/journal.pone.0271108)
Supplement: S4 Table — (PDF) [file pone.0271108.s004.pdf]

S4 Table: Projected obesity-attributable annual costs of T2DM, chronic liver diseases and liver cancer, by sex and working age group (2020 to 2040; currency USD)

Indicator, by sex and age	2020	2025	2030	2035	2040
**Type II Diabetes, Liver cancer, Chronic liver diseases: Obesity-attributable annual healthcare costs, 95% Confidence Limits**					
Males					
20-24 years	118,497,996 [± 1,366,614]	115,921,932 [± 1,397,529]	127,098,153 [± 1,479,636]	134,274,861 [± 1,631,213]	124,669,924 [± 1,535,733]
25-29 years	227,331,768 [± 2,122,368]	229,265,364 [± 2,005,221]	220,385,180 [± 2,019,770]	231,961,590 [± 2,044,584]	229,628,663 [± 2,114,708]
30-34 years	259,220,613 [± 3,111,533]	364,109,256 [± 2,828,520]	351,471,765 [± 2,666,148]	329,677,020 [± 2,627,178]	335,901,679 [± 2,577,598]
35-39 years	348,664,016 [± 3,818,654]	463,363,842 [± 3,617,426]	577,727,839 [± 3,364,608]	554,408,429 [± 3,192,049]	538,833,208 [± 3,179,966]
40-44 years	475,640,284 [± 4,008,793]	562,335,607 [± 4,044,996]	691,872,090 [± 3,936,925]	815,936,628 [± 3,721,107]	786,908,002 [± 3,549,580]
45-49 years	412,418,871 [± 3,703,639]	658,123,509 [± 4,061,343]	749,889,639 [± 4,183,373]	882,573,608 [± 4,101,963]	1,036,210,738 [± 3,955,848]
50-54 years	264,450,629 [± 3,192,907]	545,518,282 [± 3,696,499]	806,090,732 [± 4,078,093]	901,908,848 [± 4,252,860]	1,052,493,326 [± 4,226,001]
55-59 years	167,143,603 [± 2,725,333]	356,392,239 [± 3,247,328]	643,143,411 [± 3,715,243]	932,196,744 [± 4,170,176]	1,004,928,755 [± 4,332,722]
Total males	**2,273,367,779 [± 8,834,325]**	**3,295,030,030 [± 9,172,025]**	**4,167,678,808 [± 9,387,843]**	**4,782,937,726 [± 9,491,172]**	**5,109,574,295 [± 9,405,662]**
Females					
20-24 years	97,359,426 [± 1,281,033]	85,308,110 [± 1,256,371]	89,106,608 [± 1,343,367]	88,228,294 [± 1,673,818]	78,425,763 [± 1,593,943]
25-29 years	165,738,494 [± 1,759,868]	147,504,750 [± 1,513,223]	128,314,426 [± 1,470,187]	131,981,371 [± 1,546,215]	130,042,664 [± 1,911,221]
30-34 years	150,811,138 [± 2,190,588]	219,934,552 [± 1,997,604]	188,662,084 [± 1,722,990]	163,141,937 [± 1,661,589]	166,893,090 [± 1,736,397]
35-39 years	175,697,197 [± 2,557,741]	255,902,626 [± 2,437,312]	318,422,946 [± 2,246,019]	269,400,957 [± 1,952,532]	236,554,581 [± 1,880,899]

40-44 years	251,208,545 [± 2,735,695]	298,803,485 [± 2,759,595]	379,859,903 [± 2,648,853]	437,390,297 [± 2,468,243]	365,960,936 [± 2,151,481]
45-49 years	210,614,286 [± 2,520,331]	350,184,554 [± 2,858,429]	395,758,725 [± 2,898,108]	479,032,167 [± 2,799,178]	530,678,773 [± 2,621,780]
50-54 years	114,634,108 [± 2,101,979]	266,997,865 [± 2,569,767]	421,419,971 [± 2,940,998]	464,855,613 [± 2,986,361]	548,958,878 [± 2,893,757]
55-59 years	67,242,594 [± 1,821,325]	146,484,842 [± 2,130,204]	315,696,072 [± 2,630,324]	481,458,313 [± 3,014,377]	523,950,506 [± 3,067,331]
Total females	**1,233,305,788 [± 6,136,003]**	**1,771,120,784 [± 6,381,746]**	**2,237,240,735 [± 6,554,610]**	**2,515,488,949 [± 6,610,458]**	**2,581,465,192 [± 6,483,149]**
**TOTAL**	**3,506,673,566 [± 10,756,200]**	**5,066,150,815 [± 11,173,751]**	**6,404,919,544 [± 11,449,651]**	**7,298,426,675 [± 11,566,352]**	**7,691,039,487 [± 11,423,559]**

Type II Diabetes: Obesity-attributable annual healthcare costs, 95% Confidence Limits

Males					
20-24 years	93,290,658 [± 637,346]	91,654,729 [± 644,888]	100,682,695 [± 677,540]	106,636,673 [± 741,181]	99,392,366 [± 691,753]
25-29 years	156,449,081 [± 1,034,326]	166,798,320 [± 922,276]	161,187,463 [± 923,749]	169,950,061 [± 926,978]	168,669,223 [± 951,882]
30-34 years	152,850,250 [± 1,520,903]	234,901,704 [± 1,311,768]	238,074,122 [± 1,174,248]	224,578,370 [± 1,152,712]	229,526,373 [± 1,121,831]
35-39 years	187,323,411 [± 1,845,240]	265,767,368 [± 1,714,248]	354,266,164 [± 1,525,452]	352,151,787 [± 1,395,564]	342,915,115 [± 1,389,188]
40-44 years	273,421,882 [± 1,927,908]	319,564,625 [± 1,945,190]	407,555,778 [± 1,867,140]	502,055,409 [± 1,706,132]	496,761,522 [± 1,591,729]
45-49 years	269,414,283 [± 1,804,808]	412,756,971 [± 1,988,381]	461,376,347 [± 2,051,075]	553,007,049 [± 1,995,406]	666,109,361 [± 1,877,204]
50-54 years	197,062,485 [± 1,588,738]	381,256,867 [± 1,840,685]	540,993,943 [± 2,043,480]	593,552,634 [± 2,136,924]	696,857,996 [± 2,115,382]
55-59 years	130,007,236 [± 1,377,541]	267,771,488 [± 1,630,549]	459,017,296 [± 1,869,243]	641,794,214 [± 2,111,188]	678,946,983 [± 2,196,963]
Total males	**1,459,819,285 [± 4,311,608]**	**2,140,472,072 [± 4,439,605]**	**2,723,153,809 [± 4,514,558]**	**3,143,726,196 [± 4,538,840]**	**3,379,178,939 [± 4,464,520]**
Females					

20-24 years	76,227,420 [± 579,999]	67,706,386 [± 552,048]	71,028,971 [± 578,444]	70,204,491 [± 698,500]	62,871,059 [± 658,090]
25-29 years	116,256,568 [± 857,380]	109,601,958 [± 691,575]	96,427,646 [± 653,557]	99,908,721 [± 675,202]	98,230,479 [± 810,583]
30-34 years	95,181,500 [± 1,103,687]	148,124,630 [± 948,644]	134,282,225 [± 772,201]	117,229,703 [± 725,894]	120,992,344 [± 746,742]
35-39 years	102,954,194 [± 1,289,406]	157,754,212 [± 1,207,611]	207,141,774 [± 1,056,555]	182,655,965 [± 877,519]	161,462,296 [± 828,806]
40-44 years	153,829,747 [± 1,379,917]	180,907,277 [± 1,386,211]	236,330,677 [± 1,309,798]	282,541,076 [± 1,169,630]	244,049,180 [± 985,108]
45-49 years	138,208,061 [± 1,284,709]	223,748,746 [± 1,450,729]	249,042,544 [± 1,464,373]	306,145,870 [± 1,396,238]	348,585,822 [± 1,261,394]
50-54 years	82,616,545 [± 1,088,048]	182,951,430 [± 1,324,637]	280,121,336 [± 1,509,977]	303,732,551 [± 1,528,222]	361,648,018 [± 1,464,375]
55-59 years	52,381,069 [± 960,416]	108,265,577 [± 1,112,160]	222,305,985 [± 1,367,682]	329,118,448 [± 1,562,876]	352,383,484 [± 1,585,970]
Total females	**817,655,103 [± 3,100,498]**	**1,179,060,215 [± 3,187,325]**	**1,496,681,158 [± 3,238,037]**	**1,691,536,825 [± 3,215,015]**	**1,750,222,682 [± 3,091,265]**
**TOTAL**	**2,277,474,389 [± 5,310,655]**	**3,319,532,287 [± 5,465,266]**	**4,219,834,967 [± 5,555,728]**	**4,835,263,021 [± 5,562,139]**	**5,129,401,621 [± 5,430,272]**
**Liver cancer: Obesity-attributable annual healthcare costs, 95% Confidence Limits**					
Males					
20-24 years	3,882 [± 118,335]	-4,001 [± 129,586]	11,776 [± 121,084]	4,451 [± 149,885]	-157 [± 144,541]
25-29 years	42,178 [± 157,765]	22,768 [± 151,750]	-5,013 [± 165,749]	27,790 [± 144,867]	23,597 [± 165,422]
30-34 years	28,119 [± 207,647]	66,149 [± 191,370]	66,599 [± 187,879]	5,726 [± 194,895]	45,163 [± 173,375]
35-39 years	44,978 [± 213,386]	88,412 [± 229,011]	158,760 [± 213,732]	120,640 [± 215,411]	76,313 [± 215,961]
40-44 years	158,182 [± 234,874]	132,496 [± 214,814]	172,469 [± 237,316]	229,011 [± 234,700]	138,912 [± 220,619]

45-49 years	107,364 [± 196,353]	155,721 [± 230,005]	175,121 [± 221,778]	126,625 [± 237,002]	307,781 [± 241,517]
50-54 years	270,335 [± 232,773]	407,943 [± 274,201]	540,174 [± 317,317]	583,503 [± 320,838]	528,767 [± 334,683]
55-59 years	285,434 [± 224,275]	442,068 [± 278,560]	614,798 [± 329,478]	690,260 [± 367,262]	594,445 [± 389,525]
Total males	**940,472 [± 570,743]**	**1,311,557 [± 617,024]**	**1,734,681 [± 661,713]**	**1,788,006 [± 690,587]**	**1,714,822 [± 704,060]**
Females					
20-24 years	15,157 [± 106,917]	11,483 [± 110,247]	7,631 [± 110,284]	32,464 [± 146,985]	15,414 [± 138,058]
25-29 years	29,783 [± 106,120]	15,876 [± 108,402]	7,843 [± 113,011]	11,696 [± 109,815]	27,616 [± 140,592]
30-34 years	4,670 [± 104,740]	30,882 [± 100,851]	12,424 [± 105,029]	8,100 [± 110,861]	8,063 [± 107,501]
35-39 years	-4,803 [± 104,271]	4,693 [± 96,882]	26,731 [± 98,394]	12,607 [± 97,651]	8,234 [± 106,732]
40-44 years	68,350 [± 81,009]	48,690 [± 93,528]	47,204 [± 94,147]	71,851 [± 90,434]	33,850 [± 68,473]
45-49 years	118,869 [± 110,985]	174,184 [± 140,668]	127,702 [± 149,884]	136,002 [± 147,532]	148,695 [± 147,659]
50-54 years	130,334 [± 122,775]	142,958 [± 145,635]	223,779 [± 184,236]	234,942 [± 188,914]	198,725 [± 183,944]
55-59 years	172,430 [± 137,794]	250,409 [± 167,152]	447,703 [± 219,841]	539,169 [± 255,766]	519,734 [± 272,558]
Total females	**534,790 [± 312,212]**	**679,176 [± 348,061]**	**901,016 [± 399,059]**	**1,046,831 [± 431,901]**	**960,331 [± 443,159]**
**TOTAL**	**1,475,262 [± 650,556]**	**1,990,733 [± 708,424]**	**2,635,698 [± 772,730]**	**2,834,837 [± 814,524]**	**2,675,153 [± 831,919]**
**Chronic liver diseases: Obesity-attributable annual healthcare costs, 95% Confidence Limits**					
Males					
20-24 years	25,203,456 [± 1,203,088]	24,271,204 [± 1,233,050]	26,403,682 [± 1,309,809]	27,633,737 [± 1,445,352]	25,277,716 [± 1,363,475]

25-29 years	70,840,509 [± 1,846,544]	62,444,276 [± 1,774,060]	59,202,730 [± 1,788,487]	61,983,738 [± 1,816,604]	60,935,842 [± 1,881,102]
30-34 years	106,342,244 [± 2,706,543]	129,141,403 [± 2,498,633]	113,331,044 [± 2,386,250]	105,092,924 [± 2,352,730]	106,330,143 [± 2,314,184]
35-39 years	161,295,627 [± 3,336,417]	197,508,062 [± 3,177,213]	223,302,915 [± 2,991,305]	202,136,002 [± 2,862,722]	195,841,780 [± 2,852,315]
40-44 years	202,060,220 [± 3,506,911]	242,638,485 [± 3,540,069]	284,143,842 [± 3,457,868]	313,652,208 [± 3,298,586]	290,007,568 [± 3,165,003]
45-49 years	142,897,223 [± 3,228,166]	245,210,817 [± 3,533,828]	288,338,171 [± 3,639,302]	329,439,934 [± 3,576,071]	369,793,595 [± 3,473,688]
50-54 years	67,117,809 [± 2,759,779]	163,853,472 [± 3,193,869]	264,556,614 [± 3,514,875]	307,772,711 [± 3,662,981]	355,106,563 [± 3,643,107]
55-59 years	36,850,933 [± 2,340,838]	88,178,683 [± 2,794,432]	183,511,318 [± 3,193,807]	289,712,271 [± 3,577,481]	325,387,327 [± 3,714,041]
Total males	**812,608,022 [± 7,689,576]**	**1,153,246,402 [± 8,002,201]**	**1,442,790,318 [± 8,204,419]**	**1,637,423,525 [± 8,306,887]**	**1,728,680,534 [± 8,248,566]**
Females					
20-24 years	21,116,849 [± 1,137,196]	17,590,241 [± 1,123,190]	18,070,005 [± 1,207,426]	17,991,339 [± 1,513,988]	15,539,290 [± 1,445,169]
25-29 years	49,452,142 [± 1,533,224]	37,886,916 [± 1,341,573]	31,878,936 [± 1,312,076]	32,060,954 [± 1,386,660]	31,784,570 [± 1,725,095]
30-34 years	55,624,968 [± 1,889,333]	71,779,040 [± 1,755,086]	54,367,435 [± 1,536,675]	45,904,134 [± 1,490,525]	45,892,683 [± 1,563,935]
35-39 years	72,747,806 [± 2,206,490]	98,143,720 [± 2,114,895]	111,254,441 [± 1,979,549]	86,732,386 [± 1,741,495]	75,084,050 [± 1,685,072]
40-44 years	97,310,448 [± 2,360,782]	117,847,518 [± 2,384,332]	143,482,022 [± 2,300,432]	154,777,370 [± 2,171,638]	121,877,907 [± 1,911,477]
45-49 years	72,287,356 [± 2,165,474]	126,261,624 [± 2,458,905]	146,588,480 [± 2,496,433]	172,750,294 [± 2,421,601]	181,944,256 [± 2,293,646]
50-54 years	31,887,230 [± 1,794,267]	83,903,478 [± 2,197,233]	141,074,856 [± 2,517,042]	160,888,120 [± 2,558,750]	187,112,135 [± 2,489,096]
55-59 years	14,689,095 [± 1,541,375]	37,968,856 [± 1,809,124]	92,942,385 [± 2,236,005]	151,800,696 [± 2,564,854]	171,047,287 [± 2,611,307]

Total females	**415,115,894 [± 5,285,827]**	**591,381,393 [± 5,517,834]**	**739,658,561 [± 5,684,961]**	**822,905,293 [± 5,759,799]**	**830,282,178 [± 5,681,453]**
**TOTAL**	**1,227,723,916 [± 9,331,107]**	**1,744,627,795 [± 9,720,171]**	**2,182,448,879 [± 9,981,547]**	**2,460,328,818 [± 10,108,395]**	**2,558,962,712 [± 10,015,875]**
