# Supplementary material for: The future health and economic burden of obesity-attributable type 2 diabetes and liver disease among the working-age population in Saudi Arabia
Source: PLoS One. 2022 Jul 14;17(7):e0271108. doi: 10.1371/journal.pone.0271108 (PMC9282435; doi:10.1371/journal.pone.0271108)
Supplement: S5 Table — (PDF) [file pone.0271108.s005.pdf]

S5 Table: Projected WHO obesity categories, by sex and working age group (2020 to 2040)

Indicator, by sex and age	2020	2025	2030	2035	2040
**Healthy weight (BMI < 25): % prevalence, 95% confidence limit***					
Males					
20-34 years	47.38% [± 0.03%]	47.91% [± 0.03%]	48.38% [± 0.03%]	48.80% [± 0.03%]	49.21% [± 0.03%]
35-59 years	19.47% [± 0.02%]	18.30% [± 0.02%]	17.65% [± 0.02%]	17.02% [± 0.02%]	15.40% [± 0.02%]
Total males	**29.50% [± 0.01%]**	**28.82% [± 0.01%]**	**28.92% [± 0.01%]**	**29.56% [± 0.01%]**	**29.95% [± 0.01%]**
Females					
20-34 years	49.01% [± 0.03%]	50.33% [± 0.03%]	51.44% [± 0.03%]	52.33% [± 0.03%]	53.02% [± 0.03%]
35-59 years	19.71% [± 0.02%]	21.34% [± 0.02%]	22.74% [± 0.02%]	24.20% [± 0.02%]	26.17% [± 0.02%]
Total females	**33.47% [± 0.02%]**	**33.71% [± 0.02%]**	**34.35% [± 0.02%]**	**35.22% [± 0.02%]**	**36.68% [± 0.02%]**
**TOTAL**	**31.11% [± 0.01%]**	**30.91% [± 0.01%]**	**31.37% [± 0.01%]**	**32.18% [± 0.01%]**	**33.10% [± 0.01%]**
**Pre-obese (BMI ≥ 25 and < 30): % prevalence, 95% confidence limit***					
Males					
20-34 years	32.84% [± 0.02%]	33.48% [± 0.03%]	34.13% [± 0.03%]	34.74% [± 0.03%]	35.32% [± 0.02%]
35-59 years	36.71% [± 0.02%]	34.75% [± 0.02%]	32.59% [± 0.02%]	30.41% [± 0.02%]	28.64% [± 0.02%]
Total males	**35.31% [± 0.02%]**	**34.30% [± 0.02%]**	**33.16% [± 0.02%]**	**32.12% [± 0.02%]**	**31.51% [± 0.02%]**
Females					
20-34 years	30.97% [± 0.03%]	32.45% [± 0.03%]	33.81% [± 0.03%]	35.09% [± 0.03%]	36.29% [± 0.03%]
35-59 years	30.21% [± 0.02%]	30.70% [± 0.02%]	31.26% [± 0.02%]	31.77% [± 0.02%]	32.05% [± 0.02%]

Total females	**30.57% [± 0.02%]**	**31.45% [± 0.02%]**	**32.29% [± 0.02%]**	**33.07% [± 0.02%]**	**33.71% [± 0.02%]**
**TOTAL**	**33.40% [± 0.01%]**	**33.08% [± 0.01%]**	**32.76% [± 0.01%]**	**32.56% [± 0.01%]**	**32.54% [± 0.01%]**
**Obese (BMI ≥ 30): % prevalence, 95% confidence limit***					
Males					
20-34 years	19.78% [± 0.02%]	18.61% [± 0.02%]	17.49% [± 0.02%]	16.45% [± 0.02%]	15.48% [± 0.02%]
35-59 years	43.83% [± 0.02%]	46.95% [± 0.02%]	49.76% [± 0.02%]	52.56% [± 0.02%]	55.96% [± 0.02%]
Total males	**35.18% [± 0.01%]**	**36.88% [± 0.01%]**	**37.92% [± 0.01%]**	**38.31% [± 0.01%]**	**38.54% [± 0.01%]**
Females					
20-34 years	20.02% [± 0.02%]	17.22% [± 0.02%]	14.75% [± 0.02%]	12.58% [± 0.02%]	10.70% [± 0.02%]
35-59 years	50.08% [± 0.03%]	47.96% [± 0.02%]	46.00% [± 0.02%]	44.03% [± 0.02%]	41.79% [± 0.02%]
Total females	**35.96% [± 0.02%]**	**34.85% [± 0.02%]**	**33.36% [± 0.02%]**	**31.71% [± 0.02%]**	**29.61% [± 0.02%]**
**TOTAL**	**35.50% [± 0.01%]**	**36.01% [± 0.01%]**	**35.86% [± 0.01%]**	**35.26% [± 0.01%]**	**34.36% [± 0.01%]**

* Note that confidence limits represent Monte Carlo error from the microsimulation, and do not reflect uncertainty in the non-linear regression model used to project BMI trends.
